# Supplementary material for: Modeling flexible behavior in childhood to adulthood shows age-dependent learning mechanisms and less optimal learning in autism in each age group
Source: PLoS Biol. 2020 Oct 27;18(10):e3000908. doi: 10.1371/journal.pbio.3000908 (PMC7591042; doi:10.1371/journal.pbio.3000908)
Supplement: S1 Text — (DOCX) [file pbio.3000908.s002.docx]

# Modeling flexible behavior in childhood to adulthood shows age-dependent learning mechanisms and less optimal learning in autism in each age group

## S1 Text: Supplementary Methods

## Additional sample information

EU-AIMS Longitudinal European Autism Project (LEAP) is a multi-site study within the EU-AIMS clinical research program [1, 2]. Participants were recruited between January 2014 and March 2017 from six European sites: King’s College London (IoPPN/KCL, UK), Autism Research Centre, University of Cambridge (UCAM, UK), University Medical Centre Utrecht (UMCU, Netherlands), Radboud University Nijmegen Medical Centre (RUNMC, Netherlands), Central Institute of Mental Health (CIMH, Germany) and the University Campus Bio-Medico (UCBM) in Rome, Italy. Participants were recruited from existing volunteer databases and research cohorts, local outpatient centers, special educational needs schools, mainstream schools, community centers and posts on special educational needs forums. Further details regarding ethnicity, education and income level is reported in [3].

For ASD individuals, inclusion criteria were a clinical diagnosis of ASD according to DSM-IV, DSM-IV-TR, DSM-V or ICD-10 and aged between 6 and 30 years. Confirmation of each individual’s clinical diagnosis was collected at each site (e.g. from psychiatrist/psychologist or pediatrician). Exclusion criteria included psychosis and bipolar disorder, a history of alcohol or substance abuse or dependence in the past year and significant hearing and visual impairments not corrected by glasses or hearing aids. Where there was medication use, individuals were asked that this be stable for 8 weeks at the time of their first visit. For TD individuals, the same criteria applied except for all psychiatric and neurodevelopmental disorders served as exclusion criteria here. Further information concerning additional inclusion/exclusion criteria relevant to other study modalities (e.g. MRI) is reported in [3].

## Task Instructions

The task instructions were taken from [4] and given in each site’s language.

“On the screen two colored patterns are presented: one yellow, one blue. One of these colors is correct more often than the other one and the computer will tell you whether your choice was correct or incorrect. Choose the color which tends to be correct more often. You have to find out by trial and error which color that is. On certain moments the rule can change, i.e. the other color is now correct more often. Then, switch your response to that color. This can happen one or more times during the task.”

Each trial required a response for the task to continue and each emoticon (happy/sad) was accompanied by a matching sound.

## Model specifications

### *Standard Rescorla-Wagner model*

In the simple Rescorla-Wagner (RW) model, stimuli acquire value when there is a mismatch between prediction and outcome, known as the prediction error. Unlike the counterfactual update (CU) model, the RW model only updates the value of the correct choice stimuli.


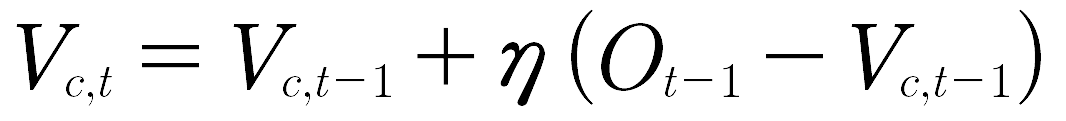


Here, the value *V* of the chosen stimuli *c* is updated with the prediction error. *O* is the outcome received. The learning rate *η* denotes the speed of learning.

*Effective number of parameters*

Although it appears that the number of parameters of the RW and CU models are identical, the additional equation in the CU model actually changes the joint parameter space, resulting in more effective number of parameters than the RW model (see Table S2).

*Model learning rates*

Whether it is necessary to separately quantify learning rates for the counterfactual update is an empirical question that can be answered using model comparison. When performing this model comparison, the model with a single learning rate was favored (ΔLOOIC > 100). In line with this, the estimated learning rates from the dual learning rate model are highly correlated (r > 0.95, see Figure S7), and the posterior distributions of the two learning rates largely overlap (95% HDI of the difference credibly contains 0), suggesting that indeed counterfactual learning occurs at the same rate as the ‘normal’ learning.

### *More information on value sensitivity*

Commonly, *β* in the Softmax function depicts the inverse temperate. If *β* is low, the choice probabilities of option A and B are not that discrepant, leading to more “randomness” in one’s choice behavior. If *β* is high, the choice probabilities will be more unbalanced and choice behavior will be more deterministic. Thus, *β* can be thought of as sensitivity to the value difference (the difference between the values of the two choice options). We refer to *β* in this paper as the value sensitivity, as it reflects a sensitivity to the values computed by the model. Previous literature has used the term choice variability to convey the modulatory impact on choice that the parameter provides [5] (see also [6]).

### *Model fitting*

Hierarchical Bayesian Analysis is considered superior to other modeling methods such as maximum likelihood estimation as it: (1) allows for individual differences whilst pooling information across individuals via a hyper-parameter and, (2) finds full posterior distributions for both group-level parameters and individual-level parameters [7]. Posterior inference was performed using Markov Chain Monte Carlo (MCMC) sampling within RStan. Four MCMC chains were used, with 1,000 post-warmup iterations per chain, resulting in 4,000 valid MCMC samples. Model convergence was assessed by examining Rhat values, an index of the convergence of the chains [8]. Rhat values of all models were lower than 1.1 suggesting MCMC samples were well mixed and converged to stationary distributions. Higher Bayesian model weights denote better model fit. Model parameters were then extracted for each model that provided the best estimate within a group (here defined as > 0.50 model weight).

### *Parameter specification*

Following the approach in the “hBayesDM” package ([9]), we assumed, for instance, that a generic individual-level parameter *ϕ* was drawn from a group-level normal distribution, namely, *ϕ* ~ Normal (*μ_ϕ_*, *σ_ϕ_*), with *μ_ϕ_* and *σ_ϕ_*. being the group-level mean and standard deviation, respectively. Both these group-level parameters were specified with weakly-informative priors [10]: *μ_ϕ_* ~ Normal (0, 1) and *σ_ϕ_*.~ half-Cauchy (0, 5). This was to ensure that the MCMC sampler traveled over a sufficiently wide range to sample the entire parameter space. Parameters were constrained for learning rate *η/θ* and experience decay *ρ* (both [0 1] constraint, with inverse probit transform), softmax’s indecision point *α* ([−0.5 0.5] constraint, with inverse probit transform), and softmax inverse temperature *β* ([0 5] constraint, with inverse probit transform).

*Model validation*

Once the winning models for each diagnosis and age group were established, we used the one-step-ahead prediction method to examine absolute model fit (e.g. [11]). This method is a post-hoc approach that moves beyond relative model fit to validate that the models capture task behavior effectively. Parameters are drawn from the joint posterior distribution in combination with the outcome sequence to predict future trials. The observed choices and outcomes are then used to update the action probabilities. The prediction was repeated 4,000 times (per trial per participant) and action probabilities were averaged over these repetitions.

*Model recovery*

Following establishment of the winning models, we conducted model recovery analyses using simulated data from 40 participants. The basic Rescorla-Wagner model was omitted as all other models consistently outperformed it. Results showed that all models’ identity can be well recovered (Figure S4).

*Simulations of optimal learning rates*

We ran simulations to understand the optimal learning rates for each model, taking values for the inverse temperature parameter (‘value sensitivity’) reflective of each age group’s mean value. For each simulation, we took a small grid per parameter (0:0.01:1), and we repeat this simulation for 500 times. We then looked for the parameter(s) that gave the highest choice accuracy (choosing the more rewarded stimulus).

The R-P simulation shows a reward learning rate higher than the punishment learning rate is optimal (Figure 3D). In order to corroborate this and in light of our findings, we ran a simulation to substantiate the indication that when punishment learning rates are comparable, a higher reward learning rate results in quicker value update for the correct choice option and thus the value difference (difference between the chosen and unchosen options) is larger, relative to a lower reward learning rate. In other words, it distinguishes the two choice options quicker when the reward learning rate is high, assuming comparable punishment learning rates (Figure S5). These findings contribute to continued debates within the literature regarding higher learning rates for positive value compared to negative value stimuli (see also [12-14]).

**References**

1. Loth E, Spooren W, Murphy DG. New treatment targets for autism spectrum disorders: EU-AIMS. The Lancet Psychiatry. 2014;1(6):413-5. doi: 10.1016/S2215-0366(14)00004-2.

2. Murphy D, Spooren W. EU-AIMS: a boost to autism research. Nature reviews Drug discovery. 2012;11(11):815-6. Epub 2012/11/06. doi: 10.1038/nrd3881. PubMed PMID: 23123927.

3. Charman T, Loth E, Tillmann J, Crawley D, Wooldridge C, Goyard D, et al. The EU-AIMS Longitudinal European Autism Project (LEAP): clinical characterisation. Molecular autism. 2017;8:27. Epub 2017/06/27. doi: 10.1186/s13229-017-0145-9. PubMed PMID: 28649313; PubMed Central PMCID: PMCPMC5481972.

4. den Ouden HE, Daw ND, Fernandez G, Elshout JA, Rijpkema M, Hoogman M, et al. Dissociable effects of dopamine and serotonin on reversal learning. Neuron. 2013;80(4):1090-100. Epub 2013/11/26. doi: 10.1016/j.neuron.2013.08.030. PubMed PMID: 24267657.

5. Robinson OJ, Chase HW. Learning and Choice in Mood Disorders: Searching for the Computational Parameters of Anhedonia. Computational Psychiatry. 2017;1:208-33. doi: 10.1162/CPSY_a_00009.

6. Huys QJ, Pizzagalli DA, Bogdan R, Dayan P. Mapping anhedonia onto reinforcement learning: a behavioural meta-analysis. Biology of mood & anxiety disorders. 2013;3(1):12-. doi: 10.1186/2045-5380-3-12. PubMed PMID: 23782813.

7. Ahn WY, Krawitz A, Kim W, Busmeyer JR, Brown JW. A Model-Based fMRI Analysis with Hierarchical Bayesian Parameter Estimation. Journal of neuroscience, psychology, and economics. 2011;4(2):95-110. doi: 10.1037/a0020684. PubMed PMID: 23795233; PubMed Central PMCID: PMC3686299.

8. Gelman A, Rubin DB. Inference from Iterative Simulation Using Multiple Sequences. Statist Sci. 1992;7(4):457-72. doi: 10.1214/ss/1177011136.

9. Ahn WY, Haines N, Zhang L. Revealing Neurocomputational Mechanisms of Reinforcement Learning and Decision-Making With the hBayesDM Package. Computational Psychiatry. 2017;1:24-57. Epub 2018/03/31. doi: 10.1162/CPSY_a_00002. PubMed PMID: 29601060; PubMed Central PMCID: PMCPMC5869013.

10. Gelman A, Carlin JB, Stern HS, Dunson DB, Vehtari A, Rubin DB. Bayesian Data Analysis, Third Edition: Taylor & Francis; 2013.

11. Swart JC, Frobose MI, Cook JL, Geurts DE, Frank MJ, Cools R, et al. Catecholaminergic challenge uncovers distinct Pavlovian and instrumental mechanisms of motivated (in)action. Elife. 2017;6. Epub 2017/05/16. doi: 10.7554/eLife.22169. PubMed PMID: 28504638; PubMed Central PMCID: PMCPMC5432212.

12. Gershman SJ. Do learning rates adapt to the distribution of rewards? Psychonomic bulletin & review. 2015;22(5):1320-7. Epub 2015/01/15. doi: 10.3758/s13423-014-0790-3. PubMed PMID: 25582684.

13. Lefebvre G, Lebreton M, Meyniel F, Bourgeois-Gironde S, Palminteri S. Behavioural and neural characterization of optimistic reinforcement learning. Nature Human Behaviour. 2017;1(4). doi: 10.1038/s41562-017-0067.

14. Van Slooten JC, Jahfari S, Knapen T, Theeuwes J. How pupil responses track value-based decision-making during and after reinforcement learning. PLoS computational biology. 2018;14(11):e1006632. doi: 10.1371/journal.pcbi.1006632.
