# Supplementary material for: Modeling flexible behavior in childhood to adulthood shows age-dependent learning mechanisms and less optimal learning in autism in each age group
Source: PLoS Biol. 2020 Oct 27;18(10):e3000908. doi: 10.1371/journal.pbio.3000908 (PMC7591042; doi:10.1371/journal.pbio.3000908)
Supplement: S2 Text — (DOCX) [file pbio.3000908.s003.docx]

# Modeling flexible behavior in childhood to adulthood shows age-dependent learning mechanisms and less optimal learning in autism in each age group

## S2 Text: Additional IQ and subsample analyses

We examined the influence of IQ on task behavioral analyses reported in the main article in two ways: (1) using IQ as a confound regressor, and (2) examining task behavior in an IQ-matched subsample. We also examined models runs using IQ as a covariate after z-transform (3).

## (1) Covariate analyses – using IQ residuals

Firstly, there is a notable literature suggesting that to try and ‘control for’ or ‘covary out’ IQ in this context would be incorrect since it is known that the groups (here, TD/ASD) differ on this measure (11, 12). Particularly, perhaps, given that IQ is inextricably related to learning. We therefore repeated the linear mixed-effects models and Welch’s *t*-tests with the residuals of each dependent variable (i.e. all individual task performance measures and model parameters) and IQ regressions.

Given the correlations between IQ and task performance measures (*r*’s ranging −0.17 to 0.31), it is unlikely that the effects in the main text (and interaction for win-staying) can be accounted for by IQ. Nonetheless, we ran three simple regressions between each task performance measure and IQ and carried the residual from each one forward to the mixed-effects model. As per the results in the main text we observed the following: (1) a significant main effect of diagnosis on perseverative errors (*F_1,565.58_* = 4.19, *p* = 0.041); (2) significant main effects of both diagnosis and age group on win-staying (diagnosis: *F_1,562.90_* = 5.18, *p* = 0.023); age group: *F_1,536.19_* = 34.95, *p* = 5.4×10^−15^); (4) a significant diagnosis × age group interaction (*F_1,561.65_* = 3.32, *p* = .037) and (5) significant main effects of both diagnosis and age group on lose-shifting (diagnosis: *F_1,570_* = 7.73, *p* = .017; age group: *F_1,570_* = 22.40, *p* = 5.6×10^−10^). Again, the only significant between-diagnosis post-hoc pairwise contrast was in the adolescents (*p* = .0079).

**(2) IQ-matched subsample**

In addition to the above analyses examining IQ as a confound regressor, we conducted further post-hoc analyses with an IQ-matched subsample. Here, we used the “exact matching” method (‘MatchIt’ package), taking individuals from within an IQ range of 90-130 matching both the mean and variance. Table S3 presents IQ mean, standard deviation and range of the full sample and IQ-matched subsample, split by diagnostic group, overall and within each age group. Figure S3 presents within-age group between-diagnostic group comparisons on all four task behavioral measures (Figure S3E-H; analyses discussed below), with results from the full sample analyses presented above (Figure S3A-D) for reference. The pattern of results is largely unchanged.

Task behavioral analyses were then re-run using the IQ-matched subsample. As shown in Table S3, diagnostic groups did not differ on sex, age or IQ, either overall or within each age group (*p*s>.2). A repeated-measures analysis of accuracy showed significant main effects of phase (*F*_(1,359)_=199.99, *p*<2.2×10^−16^), diagnosis (*F*_(1,359)_=12.40, *p*=.0005) and age group (*F*_(2,359)_=21.53, *p*=1.06×10^−8^), but no significant interactions (all *p*s>.3), as in the full sample. Post-hoc analyses confirmed the same pattern as in the full sample analyses – accuracy was on average significantly higher: (i) in the acquisition phase than the reversal phase (*M*_acq_=0.79, *SD*_acq_=0.15, *M*_rev_=0.66, *SD*_rev_=0.19), (ii) in TD individuals compared to ASD individuals (*M*_TD_=0.75, *SD*_TD_=0.17, *M*_ASD_=0.69, *SD*_ASD_=0.18) and (iii) in older age groups compared to younger age groups (Adults-Adolescents, *p*=.015, *0*; Adults-Children, *p*<.0001, *d*=0.80; Adolescents-Children, *p*=.0002, *d*=0.42).

Next, the significant main effect of diagnosis on perseverative errors was also present in this subsample (*F*_(1,357.66)_=4.27, *p*=.023, *d*=0.26), such that ASD individuals made on average significantly more perseverative errors than TD individuals. As in the full sample, there was no significant effect of age nor interaction between diagnosis and age group (*p*s>.08).

Feedback sensitivity analyses also replicated the full sample findings: ASD individuals again showed on average significantly less win-stay and more lose-shift behavior relative to TD individuals, and for both there was a main effect of age (win-stay: diagnosis (*F*_(1,357.37)_=7.22, *p*=.0075, *d*=0.24), age group (*F*_(2,303.82)_=30.48, *p*=8.64×10^−13^); lose‑shift: diagnosis (*F*_(1,359)_=8.38, *p*=.0004, *d*=0.37), age group (*F*_(2,359)_=14.89, *p*=6.16×10^−7^). Pairwise post-hoc comparisons revealed win-staying increased and lose-shifting decreased with age.

Finally, for win-stay behavior, the predicted interaction between diagnosis and age group was not significant in this subsample (*p*=.2), however, the pre-planned within age-group between-diagnosis group analysis did show, as in the full sample, that ASD adolescents displayed less win-staying than TD adolescents as in the full sample analyses (*p*=0.0050, *d*=0.51). This analysis survived Bonferroni correction (correcting for task behavioral measures × age groups: *p* value =.05/(3×3)=.0056), as in the full sample analyses. For lose-shift behavior, there was no significant interaction between diagnosis and age group (*p*=.3) and no between-diagnosis group age group comparisons survived Bonferroni correction (*p*s>.015), replicating the patterns of results in the full sample findings.

In summary, analyses on an IQ-matched subsample are consistent with the full sample findings, which is in line with the observation that inclusion of IQ as a confound regressor in the full sample did not affect the pattern of results (see Figure S3).

## (3) Reinforcement learning models with IQ as a covariate

For our main analyses we did not use IQ as a covariate for the reinforcement learning models for reasons described above regarding covarying measures noted as being different in groups of interest (here, TD/ASD) and for the reason that we were interested in how the model parameters related to IQ. However, for completeness, we report here the model weights for the models by age and diagnosis group using IQ as a covariate after z-transform (Table S5).

**Risi et al. ADI-R criteria ASD subsample**

We also conducted behavioral analyses using a subset of ASD individuals with a narrower definition of ASD as specified by Risi and colleagues (2006) using the ADI-R. The criteria are as follows: meets criteria on ADI-R Social domain (A: Social reciprocity >= 10) and meets criteria on either ADI-R Communication (B: Verbal >=8) or ADI-R Behavior domain (C: Repetitive behavior >= 3). 307 of 321 ASD participants had sufficient data to calculate the Risi threshold. Of the 307 with available data, 236 met Risi criteria (77%; Table S1).

Results are, once again, largely unchanged (see Figures S2I-L, compare to Figures S2A-D). As in the full sample, diagnostic groups did not differ on sex or age, either overall or within each age group (all *p*s>.1), however, all groups differed significantly on full-scale IQ, with TD groups scoring higher than ASD groups (*p*s ranging .026 to .0001). As in the full sample findings, repeated-measures analysis of accuracy showed significant main effects of phase (*F*_(1,481)_=275.44, *p*<×10^−16^), diagnosis (*F*_(1,481)_=17.85, *p*=2.87×10^−5^) and age group (*F*_(2,481)_=13.54, *p*=1.89×10^−6^), but no significant interactions (all *p*s>.1). Post-hoc analyses again showed accuracy was on average significantly higher: (i) in the acquisition phase than the reversal phase, (ii) in TD individuals compared to ASD individuals and (iii) in older age groups compared to younger age groups (Adults-Adolescents, *p*=.018, *d*=0.28; Adults-Children, *p*<.0001, *d*=0.62; Adolescents-Children, *p*=.018, *d*=0.32).

The significant main effect of diagnosis on perseverative errors was again observed (*F*_(1,480.69)_=10.93, *p*=.0010, *d*=0.32), such that ASD individuals made on average significantly more perseverative errors than TD individuals. As before, however, there was no significant age effect nor interaction between diagnosis and age group (*p*s>.3).

Feedback sensitivity analysis also replicated the pattern of results in the full sample analyses: ASD individuals again showed on average significantly less win-stay and more lose-shift behavior relative to TD individuals, and for both there was a main effect of age (win-stay: diagnosis (*F*_(1,480.61)_=9.22, *p*=.0025, *d*=0.26), age group (*F*_(2,455.25)_=25.41, *p*=3.45×10^−11^); lose‑shift: diagnosis (*F*_(1,391.67)_=9.42, *p*=.0023, *d*=0.30), age group (*F*_(2,228.42)_=15.58, *p*=4.54×10^−7^). As before, pairwise post-hoc comparisons showed win-staying increased and lose-shifting decreased with age. In these analyses, the predicted interaction between diagnosis and age group for win-stay behavior was not significant (*p*=.1), however, pre-planned within-age group between-diagnosis group analysis showed ASD adolescents displayed less win-staying than TD adolescents, as in the full sample analyses (*p*=0.0007, *d*=0.51). As before, this survived Bonferroni correction (correcting for task behavioral measures × age groups: *p* value =.05/(3×3)=.0056). For lose-shift behavior, the pattern of results was also unchanged: there was no significant interaction between diagnosis and age group (*p*=.4) and no between-diagnosis group age group comparisons survived Bonferroni correction (*p*s>.008).

To conclude, the pattern of results is largely unchanged when examining a ‘narrower’ ASD subsample (see Figure S2).
