# Supplementary material for: Modeling flexible behavior in childhood to adulthood shows age-dependent learning mechanisms and less optimal learning in autism in each age group
Source: PLoS Biol. 2020 Oct 27;18(10):e3000908. doi: 10.1371/journal.pbio.3000908 (PMC7591042; doi:10.1371/journal.pbio.3000908)
Supplement: S3 Text — (DOCX) [file pbio.3000908.s004.docx]

# Modeling flexible behavior in childhood to adulthood shows age-dependent learning mechanisms and less optimal learning in autism in each age group

## S3 Text: Evidence of learning

In order to establish evidence of learning, we conducted supplementary analyses, similar to those described by (2). We examined whether correct acquisition and reversal choices were above chance, even where individuals did not meet the formal learning criterion. As noted by (2), a previously used learning criterion for this task is eight consecutive correct responses (10). This criterion requires an individual to ignore at least two instances of misleading negative feedback on correct responses or vice versa. This is a particularly strict criterion considering the probabilistic structure and trial-and-error nature of the task, so we assessed the evidence for learning in both those who passed (*N* = 361) and those who failed (*N* = 211) this criterion using further analyses.

Firstly, we used a repeated-measures ANOVA on the proportion of correct responses to confirm that subjects performed better than chance across both groups (pass/fail; *F_1,570_* = 368.5, *p* < 2.2×10^−16^) and phases of the task (*F_1,571_* = 324.7, *p* < 2.2×10^−16^). Post-hoc t-tests confirmed that both those who passed and those who failed the learning criterion performed above chance (passers: both phases – *t_361_* = 43.22, *p* < 2.2×10^−16^; failers: both phases – *t_211_* = 15.78, *p* < 2.2×10^−16^; Figure S3A). We then conducted a second repeated measures ANOVA to examine the effect of group and phase on the proportion of correct responses. Both main effects were significant (phase: *F_1,570_* = 265.44, *p* < 2.2×10^−16^; learning criterion attainment: *F_1,570_* = 368.50, *p* < 2.2×10^−16^). There was also a significant phase × learning criterion attainment interaction (*F_1, 570_* = 43.68, *p* = 8.893×10^−11^).

Next, given our *a priori* interest in development and diagnostic differences, we then examined the composition of the pass and fail groups. Chi-square analyses revealed both diagnostic groups and age groups were significantly different on learning criterion attainment (diagnostic group: *χ*^2^*_1,571_* = 9.43, *p* = 0.002; age group: *χ*^2^*_1,571_* = 30.05, *p* = 2.981×10^−7^). Table S4 shows the pass/fail splits and proportions by diagnosis and age groups. Figure S3B shows the proportion of correct responses for each diagnosis and age group for each phase, by pass/fail status.

We then examined the main effects of and potential interactions between learning criterion attainment, phase, age group and diagnostic group using a repeated-measures mixed-effects model. We also examined the effect of sex. Building on the first repeated-measures ANOVA (above: phase × learning criterion attainment), we first added age and diagnostic groups to the model (model 2). This significantly improved model fit (*p* = 0.0042). We then added sex to the model (model 3); this did not improve model fit (*p* = 0.9351). Finally, we added site as a random factor to the model (model 4). This model performed significantly better than model 2 (*p* = 0.02198).

All main effects in the model were significant or approaching significance (age group; *p* = 0.068), as were two two-way interactions: phase × learning criterion attainment (as before) and learning criterion attainment (LCA8) × age group (*p* = 0.029). Importantly, though, there were no significant age group × diagnosis interactions or LCA8 × diagnosis interactions.
