# Supplementary material for: Modeling flexible behavior in childhood to adulthood shows age-dependent learning mechanisms and less optimal learning in autism in each age group
Source: PLoS Biol. 2020 Oct 27;18(10):e3000908. doi: 10.1371/journal.pbio.3000908 (PMC7591042; doi:10.1371/journal.pbio.3000908)
Supplement: S4 Text — (DOCX) [file pbio.3000908.s005.docx]

# Modeling flexible behavior in childhood to adulthood shows age-dependent learning mechanisms and less optimal learning in autism in each age group

## S4 Text: Further results for comparisons of model parameter estimates

*Adolescents*

Comparing learning rate types within diagnostic groups, TD adolescents’ reward learning rate was significantly higher than their punishment learning rate (*p*=1.12×10^−6^, *d* = .58), suggesting TD adolescents are slower to learn from punishments than they are from rewards. In contrast there was no significant difference between learning rate types for ASD adolescents (*p*=.1). TD adolescents also showed significantly higher value sensitivity (*p*=1.51×10^−11^, *d* = 1.04), reflecting less chance-level behavior and a higher sensitivity to the value difference.

*Adults*

Adults did not differ on value sensitivity; however, a small but significant difference was observed for the indifference point parameter (*p*=.04, *d* = .27).

## Associations between task behavior and model parameters

*Children*

In children, win-stay behavior was positively correlated with the learning rate, though this was only significant in TD children (*r* = .44, *p*=.0002). Win-stay behavior was also positively correlated with value sensitivity, in both diagnostic groups (ASD *r* = .86, *p*<2.2×10^−16^; TD *r* = .86, *p*<2.2×10^−16^). Lose-shift behavior was positively correlated with the learning rate, however this was only significant in ASD children (*r* = .66, *p*=1.96×10^−11^). Comparatively, lose-shift behavior was negatively correlated with value sensitivity, however this was only significant in TD children (*r* = −0.54, *p*=3.68×10^−6^). Both pairs of lose-shift behavior correlations were significantly different (learning rate: *z* = 2.96, *p*=.002; value sensitivity: *z* = 2.29, *p*=.01). Perseverative errors were negatively correlated with the learning rate, however this was only significant in TD children (*r* = -0.55, *p*=2.38×10^−6^). Perseverative errors were also negatively correlated with value sensitivity, in both diagnostic groups (ASD *r* = −0.34, *p*=.0017; TD *r* = −0.47, *p*=9.06×10^−5^).

*Adolescents*

In adolescents, win-stay behavior was positively correlated with the reward learning rate in both diagnostic groups, though significantly higher in TD adolescents (ASD *r* = .49, *p*=3.24×10^−8^; TD *r* = .67, *p*=4.01×10^−13^; *z_ASD,TD_* = −1.92, *p*=.028). Win-stay behavior was also positively correlated with the punishment learning rate (ASD *r* = .29, *p*=.0015; TD *r* = .31, *p*=.0028) and strongly associated with value sensitivity (ASD *r* = .86, *p*<2.2×10^−16^; TD *r* = .81, *p*<2.2×10^−16^). Lose-shift behavior was only significantly negatively correlated with the reward learning rate in TD adolescents (TD *r* = −0.33, *p*=.0017; *z_ASD,TD_* = 2.12, *p*=0.017). Lose-shift behavior was also negatively correlated with value sensitivity, significantly more so in TD adolescents (ASD *r* = −0.52, *p*=2.69×10^−9^; TD *r* = -0.81, *p*<2.2×10^−16^; *z_ASD,TD_* = 3.85, *p*<.001). Perseverative errors were negatively correlated with the reward learning rate (ASD *r* = −0.57, *p*=2.38×10^−11^; TD *r* = −0.61, *p*=1.24×10^−10^) and the punishment learning rate, though this was only significant in TD adolescents (TD *r* = −0.42, *p*=4.11×10^−5^). In addition, perseverative errors were negatively correlated with value sensitivity (ASD *r* = −0.29, *p*=.0019; TD *r* = −0.37, *p*=.0004).

*Adults*

In adults, win-stay behavior was strongly positively associated with value sensitivity (ASD *r* = .87, *p*<2.2×10^−16^; TD *r* = .90, *p*<2.2×10^−16^) and strongly negatively associated with experience decay (ASD *r* = 0.75, *p*<2.2×10^−16^; TD *r* = 0.84, *p*<2.2×10^−16^). Lose-shift behavior was negatively correlated with the EWA learning rate (phi) – akin to positive correlation with a Rescorla-Wagner learning rate – (ASD *r* = −0.62, *p*=1.67×10^−14^; TD *r* = −0.59, *p*=1.51×10^−10^) as well as with value sensitivity (ASD *r* = −0.61, *p*=5.81×10^−14^; TD *r* = −0.66, *p*=1.49×10^−13^). Lose-shift was positively correlated with experience decay, significantly more so in TD adults (ASD *r* = .29, *p* = .0008; TD *r* = .64, *p*=2.51×10^−12^; *z_ASD,TD_* = −3.36, *p*<.001). Perseverative errors was negatively associated with value sensitivity (ASD *r* = −0.41, *p*=2.46×10^−6^; TD *r* = −0.56, *p*=3.41×10^−9^) and strongly positively associated with experience decay (ASD *r* = .80, *p*<2.2×10^−16^; TD *r* = .77, *p*<2.2×10^−16^).

**Task behavior and model parameter associations with age and IQ**

Correlations are given in Tables 3 and 4 in the main article. The only significant associations with age were found in adolescents. Win-staying was positively correlated with age in TD adolescents (*r*_90_=0.31, *p*=0.0035) and lose-shifting was negatively correlated with age in both ASD and TD adolescents (ASD *r*_114_=−0.27, *p*=0.0042; TD *r*_90_=−0.45, *p*=1.09×10^−5^). With respect to model parameters, value sensitivity was positively correlated with age in TD adolescents (*r*_90_=0.35, *p=*0.0008). These findings are supportive of adolescence as a notable period of transition.

Perseverative errors were negatively correlated with IQ in ASD adolescents (*r*_114_=−0.37, *p*=4.45×10^−5^) and ASD adults (*r*_126_=−0.38, *p*=8.96×10^−6^). Lose-shifting was also negatively correlated with IQ, but only in ASD adults (*r*_126_=−0.26, *p*=0.0033). In contrast, win-staying was positively correlated with IQ in ASD adolescents (*r*_114_=0.42, *p*=3.21×10^−6^), ASD adults (*r*_126_=0.40, *p*=2.72×10^−6^) and TD adults (*r*_97_=0.27, *p*=0.0067). With respect to model parameters, value sensitivity was positively correlated with IQ in ASD adolescents (*r*_114_=0.33, *p*=0.0002) and ASD adults (*r*_126_=0.34, *p*=8.21×10^−5^). Experience decay was negatively correlated with IQ in ASD adults (*r*_126_=−0.33, *p*=0.0001). No significant associations were found between model learning rates and IQ (all *p*s>.10). Examining symptomatology correlations with the residuals of IQ regressions for task behavioral measures and model parameters revealed all but one association remained significant: the association between perseverative errors and parent-reported ADHD Hyperactivity/Impulsivity in ASD adults (*p*=0.34).
