# Supplementary figures and images for: Modeling flexible behavior in childhood to adulthood shows age-dependent learning mechanisms and less optimal learning in autism in each age group
Source: PLoS Biol. 2020 Oct 27;18(10):e3000908. doi: 10.1371/journal.pbio.3000908 (PMC7591042; doi:10.1371/journal.pbio.3000908)

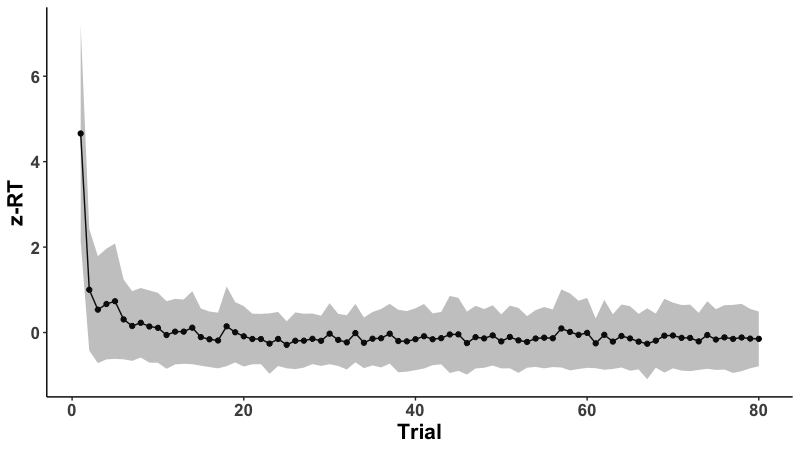

Supplement: S1 Fig — Notably, reaction times do not change at the point following reversal, illustrating that reaction times are unlikely to reflect task-relevant processes. PRL, probabilistic reversal learning; z-RT, reaction time (z-scored). (TIF) [file pbio.3000908.s006.tif]

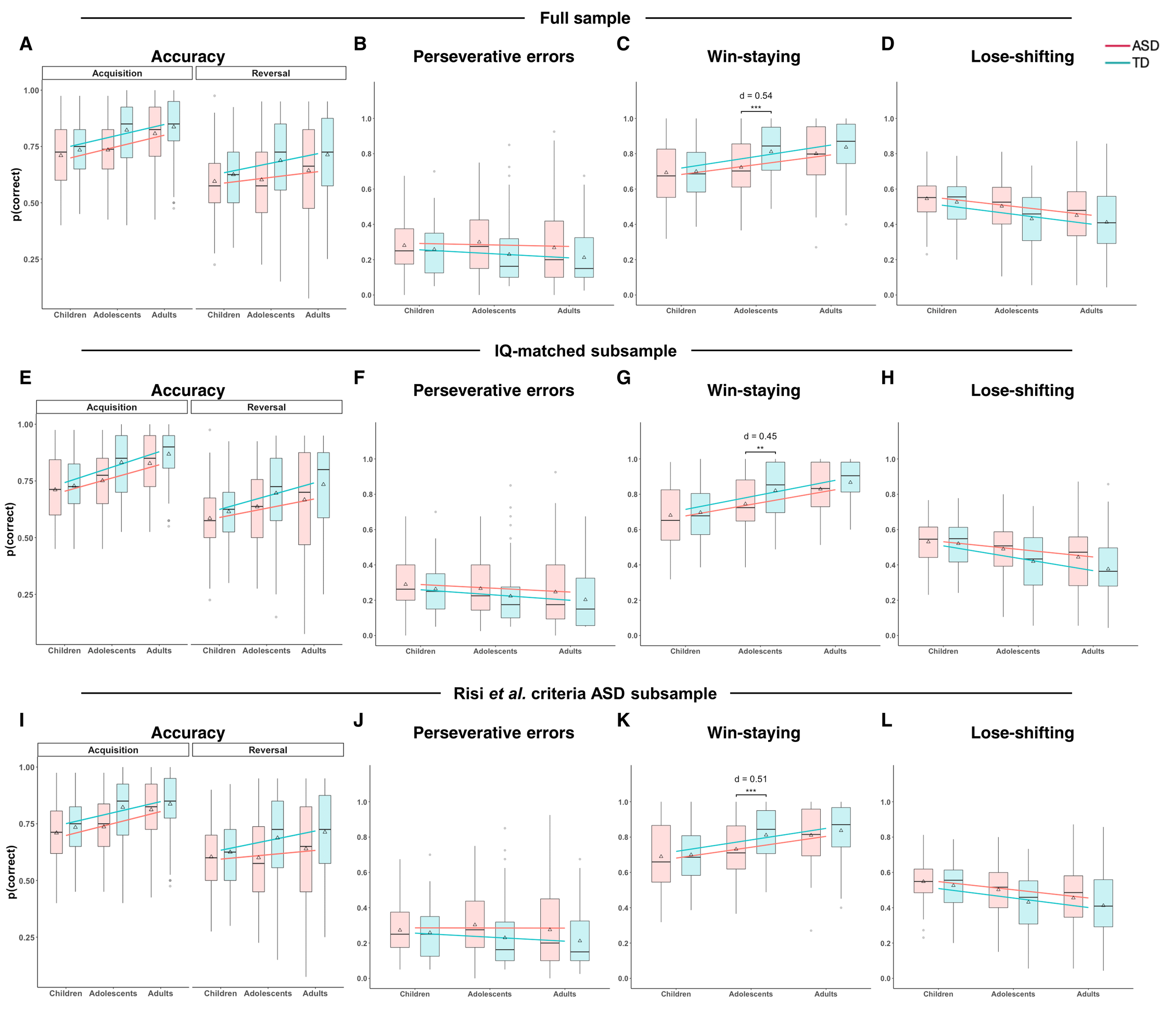

Supplement: S2 Fig — The pattern of results remains largely unchanged across both subsample analyses. ADI-R, Autism Diagnostic Interview-Revised; ASD, autism spectrum disorder. (TIF) [file pbio.3000908.s007.tif]

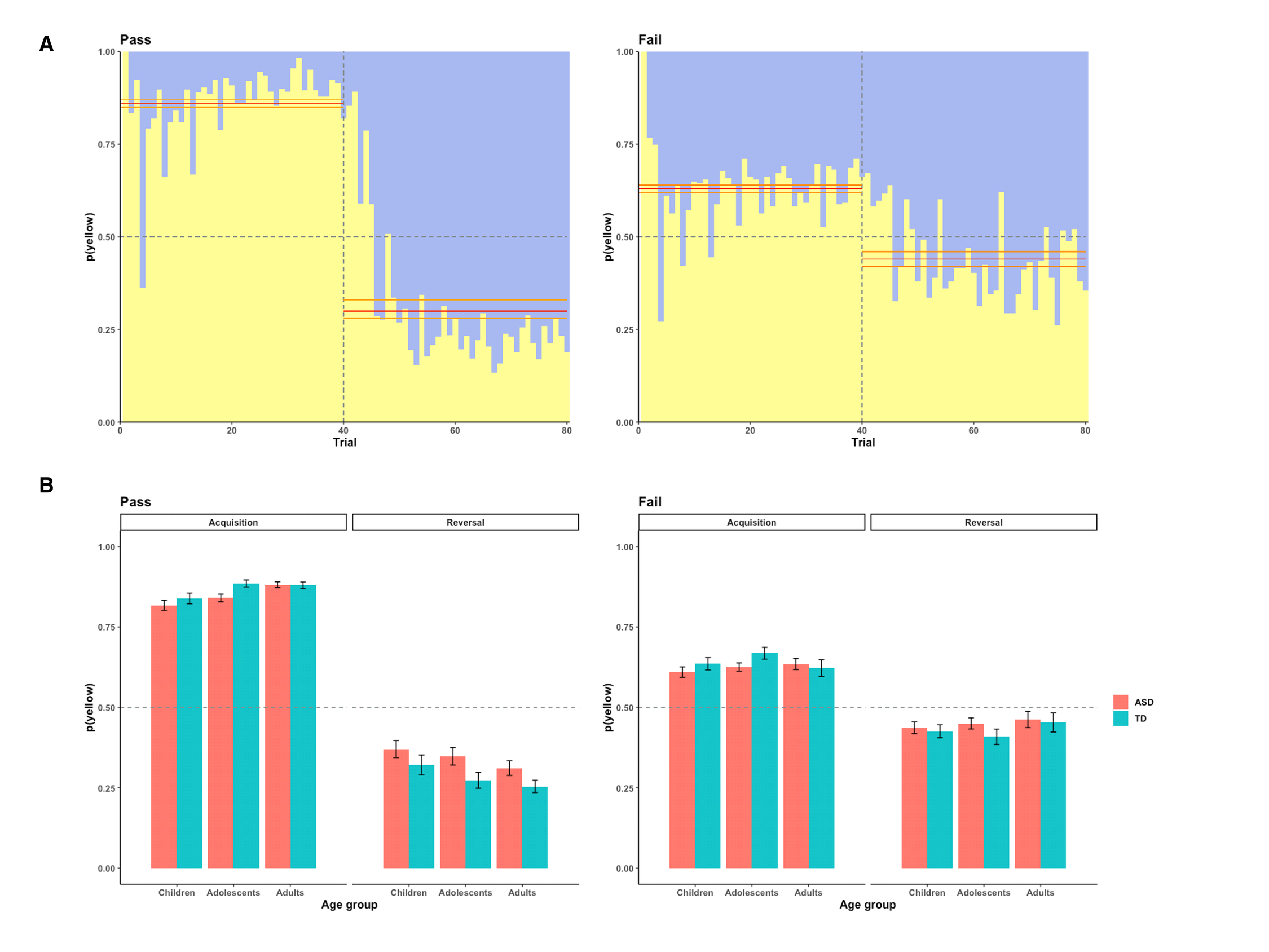

Supplement: S3 Fig — (A) Trial-by-trial average proportion of correct responses (here, yellow in acquisition phase, blue in reversal phase) plotted separately for the groups that passed and failed the learning criterion. The red lines indicate the mean for that task phase (acquisiton/reversal) and the orange lines indicate the 95% confidence intervals. Thus, both groups performed above chance in both task phases. (B) Diagnostic and age group average proportion of correct responses for each task phase, plotted separately for the pass/fail groups to confirm that perfgormance above chance was maintained even within diagnostic and age subgroups. (TIF) [file pbio.3000908.s008.tif]

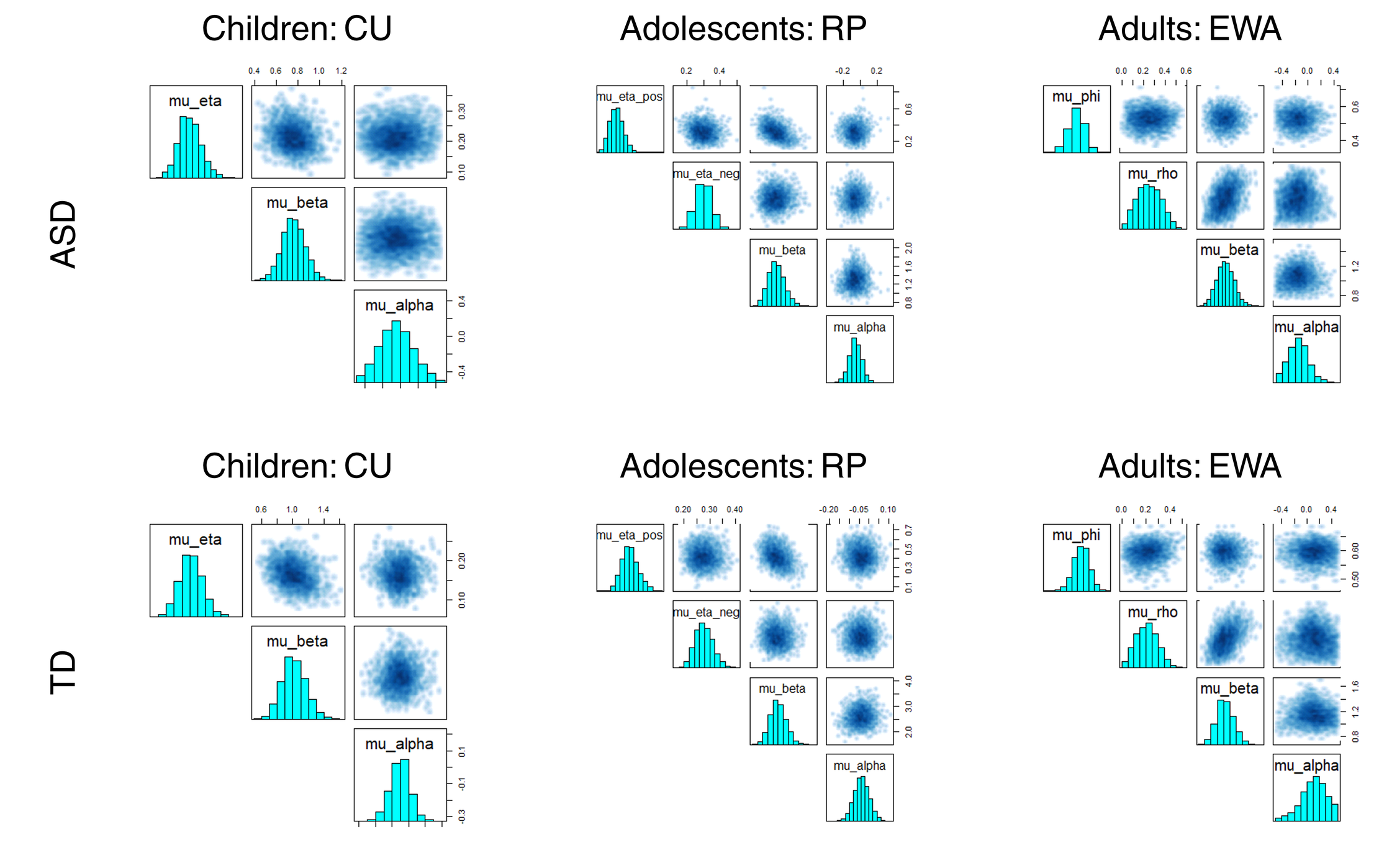

Supplement: S4 Fig — Pair plots of each group’s winning model parameters for ASD (top panel) and TD (bottom panel). In each pair plot, diagonal plots show marginal distributions of each parameter; off-diagonal plots show pairwise scatters of parameters. ASD, autism spectrum disorder; CU, counterfactual update model; EWA, experience-weighted attraction–dynamic learning rate model; RP, reward-punishment model; TD, typical development. (TIF) [file pbio.3000908.s009.tif]

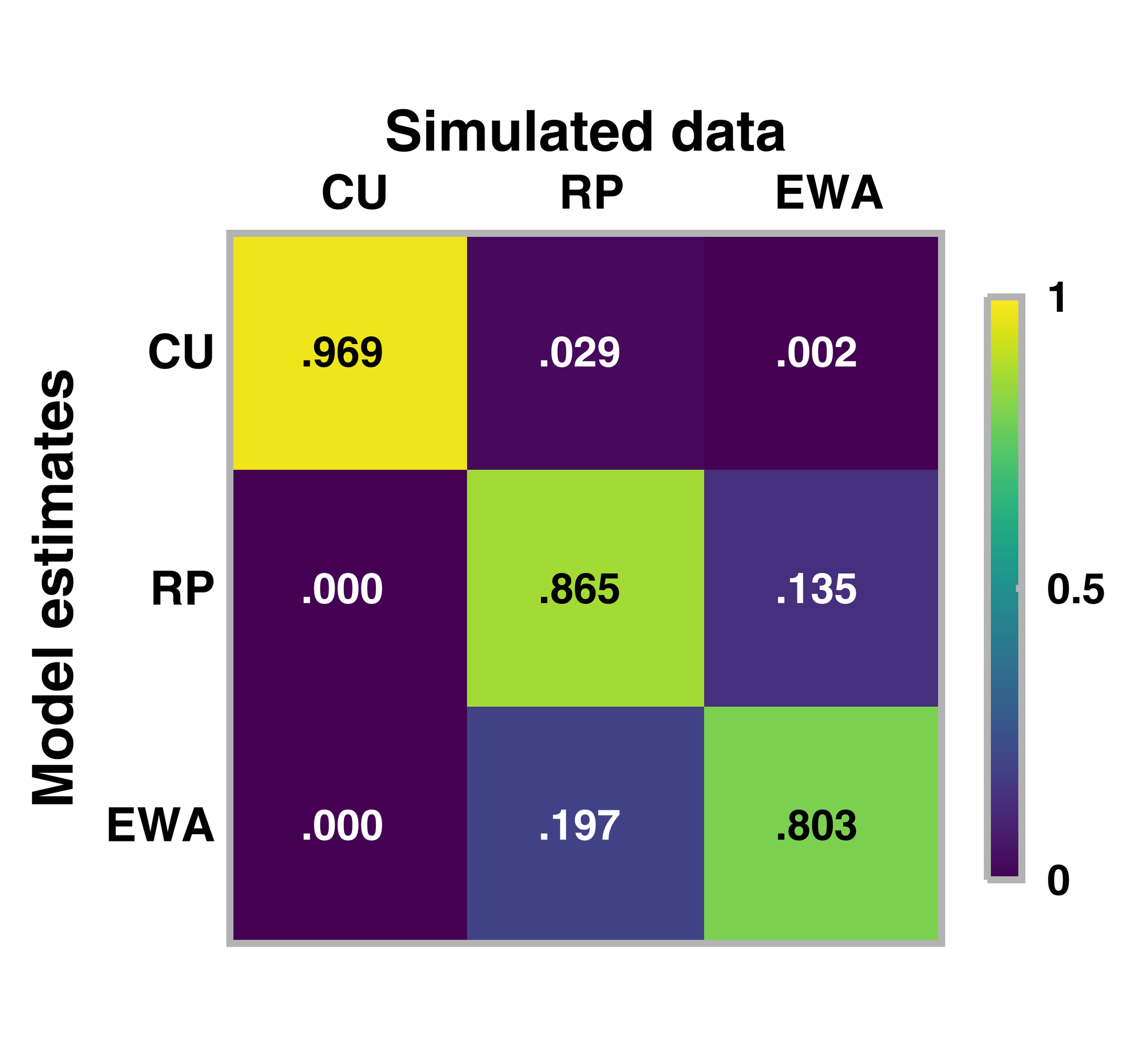

Supplement: S5 Fig — Data from 40 synthetic participants were simulated with each of our three main models. Color indicates model weights calculated with Bayesian model averaging using Bayesian bootstrap (higher model weight value indicates higher probability of the candidate model to have generated the observed data). CU, counterfactual update model; EWA, experience-weighted attraction–dynamic learning rate model; RP, reward-punishment model. (TIF) [file pbio.3000908.s010.tif]

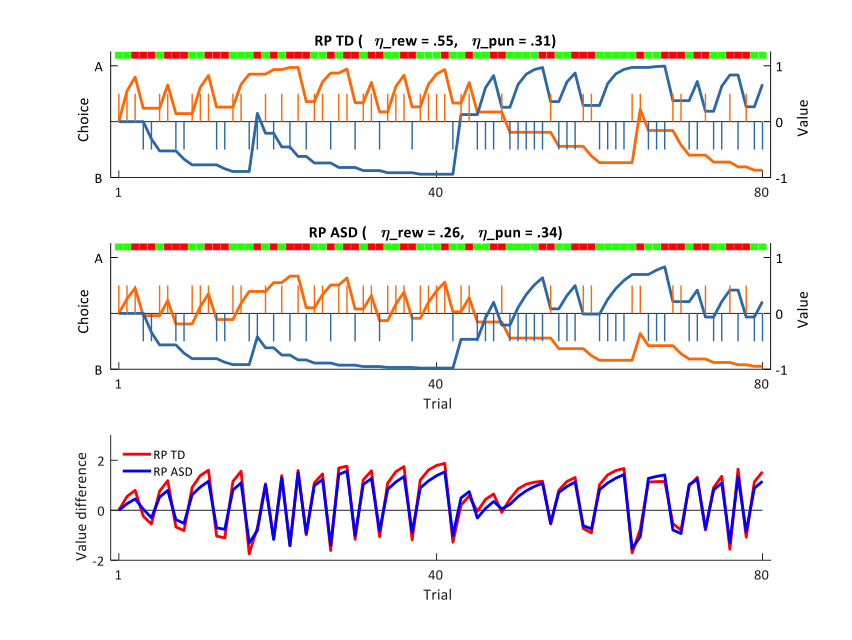

Supplement: S6 Fig — ASD, autism spectrum disorder; TD, typical development. (TIF) [file pbio.3000908.s011.tif]

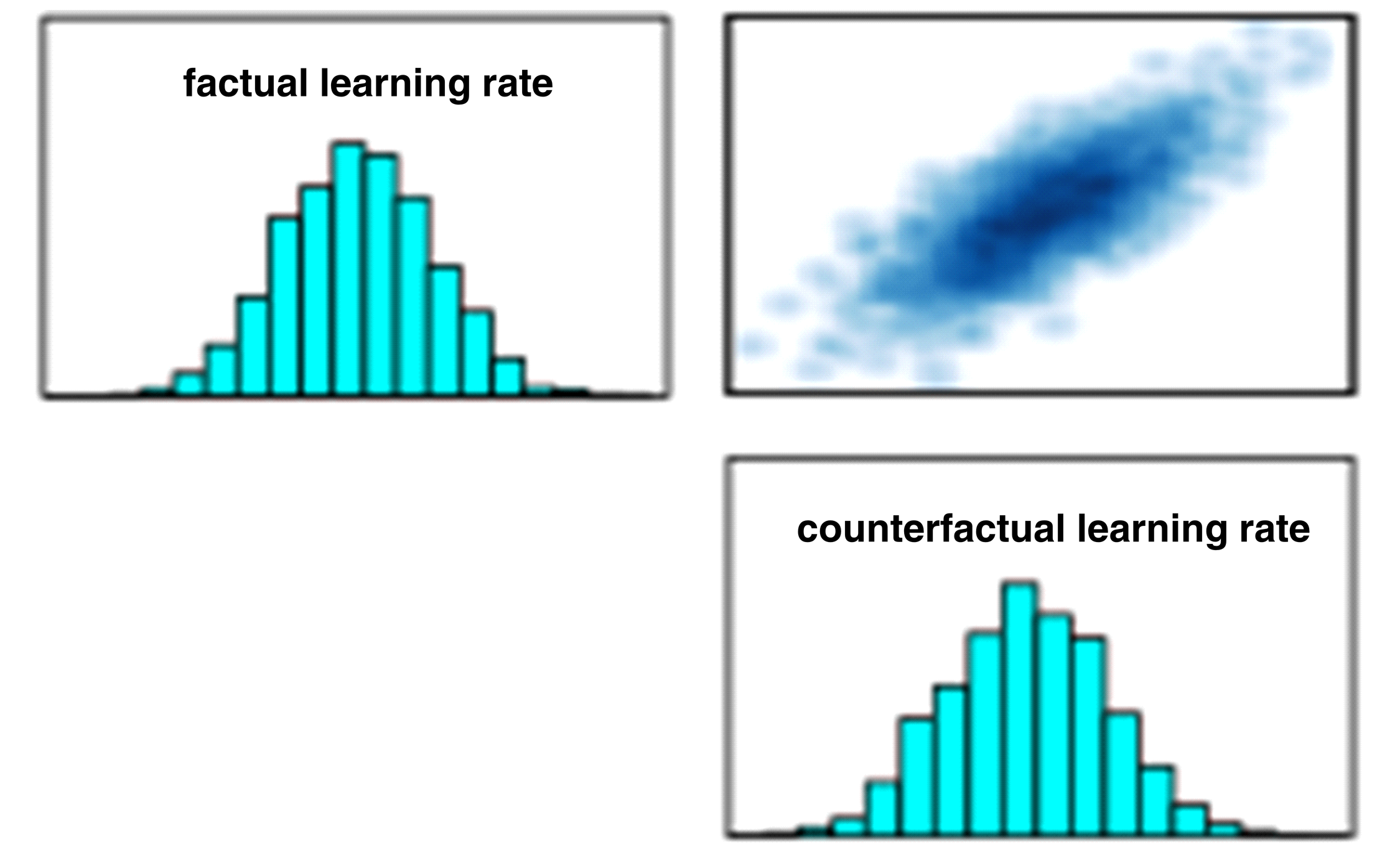

Supplement: S7 Fig — (TIF) [file pbio.3000908.s012.tif]
