# Supplementary material for: Modeling flexible behavior in childhood to adulthood shows age-dependent learning mechanisms and less optimal learning in autism in each age group
Source: PLoS Biol. 2020 Oct 27;18(10):e3000908. doi: 10.1371/journal.pbio.3000908 (PMC7591042; doi:10.1371/journal.pbio.3000908)
Supplement: S1 Table — ADI-R, Autism Diagnostic Interview-Revised; ASD, autism spectrum disorder; SD, standard deviation. (DOCX) [file pbio.3000908.s013.docx]

|  | Full ASD sample | Risi et al. ADI-R  criteria subsample |
| --- | --- | --- |
| N | 321 | 236 |
| ADI-R Social Reciprocity | 15.85 (6.81) | 18.47 (5.08) |
| ADI-R Communication | 12.81 (5.64) | 14.59 (4.71) |
| ADI-R RRB | 4.25 (2.68) | 4.70 (2.51) |
