# Supplementary material for: Modeling flexible behavior in childhood to adulthood shows age-dependent learning mechanisms and less optimal learning in autism in each age group
Source: PLoS Biol. 2020 Oct 27;18(10):e3000908. doi: 10.1371/journal.pbio.3000908 (PMC7591042; doi:10.1371/journal.pbio.3000908)
Supplement: S2 Table — CU, counterfactual update; RW, Rescorla-Wagner. (DOCX) [file pbio.3000908.s014.docx]

|  |  | RW | CW |
| --- | --- | --- | --- |
| TD | Children | 78 | 104 |
|  | Adolescents | 125 | 161 |
|  | Adults | 137 | 176 |
|  |  |  |  |
| ASD | Children | 101 | 133 |
|  | Adolescents | 154 | 204 |
|  | Adults | 200 | 246 |
