# Supplementary material for: Modeling flexible behavior in childhood to adulthood shows age-dependent learning mechanisms and less optimal learning in autism in each age group
Source: PLoS Biol. 2020 Oct 27;18(10):e3000908. doi: 10.1371/journal.pbio.3000908 (PMC7591042; doi:10.1371/journal.pbio.3000908)
Supplement: S3 Table — RBS-R, Repetitive Behavior Scale-Revised. (DOCX) [file pbio.3000908.s015.docx]

|  |  | RBS-R Stereotyped | RBS-R Self-Injurious | RBS-R Compulsive | RBS-R Ritualistic-Sameness | RBS-R Restricted Interests |
| --- | --- | --- | --- | --- | --- | --- |
| Win-stay | Children | 0.12 | -0.06 | 0.13 | -0.05 | -0.07 |
|  | Adolescents | 0.11 | 0.06 | 0.07 | 0.09 | -0.08 |
|  | Adults | -0.18 | 0.01 | -0.04 | -0.30**** | -0.22 |
| Lose-shift | Children | -0.06 | -0.21 | -0.12 | -0.17 | -0.001 |
|  | Adolescents | 0.14 | 0.05 | 0.14 | 0.18 | 0.18 |
|  | Adults | 0.24* | -0.04 | 0.13 | 0.23* | 0.11 |
| Per Errors | Children | 0.006 | 0.15 | 0.06 | 0.06 | 0.05 |
|  | Adolescents | -0.17 | -0.07 | -0.17 | -0.21** | -0.14 |
|  | Adults | 0.04 | -0.09 | 0.05 | 0.18 | 0.12 |
| Learning rate | Children | 0.14 | -0.12 | -0.14 | -0.09 | 0.05 |
|  | Adol. - reward | 0.05 | 0.08 | 0.20 | 0.11 | 0.07 |
|  | Adol. - punishment | 0.05 | 0.08 | -0.07 | 0.08 | 0.06 |
|  | Adults | -0.16 | 0.07 | -0.11 | -0.09 | -0.03 |
| Beta | Children | 0.11 | 0.07 | 0.19 | -0.11 | -0.06 |
|  | Adolescents | 0.06 | -0.03 | 0.06 | 0.10 | -0.10 |
|  | Adults | -0.28** | -0.06 | -0.09 | -0.32**** | -0.19 |
| Alpha | Children | -0.04 | -0.01 | 0.03 | -0.08 | 0.001 |
|  | Adolescents | -0.001 | -0.10 | -0.04 | 0.11 | -0.07 |
|  | Adults | -0.10 | 0.02 | -0.09 | -0.04 | -0.15 |
| Experience decay | Adults | 0.001 | -0.07 | -0.03 | 0.18 | 0.10 |

Adol. = Adolescents; **p < .01, but doesn’t survive multiple comparison corrections, ****p* < 0.0045 (Children/Adolescent Bonferroni threshold – corrected *p* value = 0.05/11 = 0.0045), **** *p* < 0.0038 (Adult Bonferroni threshold – corrected *p* value = 0.05/13 = 0.0038)
