# Supplementary material for: Modeling flexible behavior in childhood to adulthood shows age-dependent learning mechanisms and less optimal learning in autism in each age group
Source: PLoS Biol. 2020 Oct 27;18(10):e3000908. doi: 10.1371/journal.pbio.3000908 (PMC7591042; doi:10.1371/journal.pbio.3000908)
Supplement: S4 Table — IQ-m, IQ-matched subsample; SD, standard deviation. (DOCX) [file pbio.3000908.s016.docx]

|  |  | Total | | | Adults | | | Adolescents | | | Children | | |
| --- | --- | --- | --- | --- | --- | --- | --- | --- | --- | --- | --- | --- | --- |
|  |  | ASD | TD | *p* | ASD | TD | *p* | ASD | TD | *p* | ASD | TD | *p* |
| Full sample | N (% male) | 321 | 251 |  | 126 | 97 |  | 114 | 90 |  | 81 | 64 |  |
|  | Age (yrs) | 16.67  (5.92) | 16.93  (6.02) | .6 | 22.80  (3.55) | 23.25  (3.29) | .2 | 14.94  (1.71) | 15.39  (1.71) | .1 | 9.59  (1.50) | 9.52  (1.54) | .7 |
|  | IQ | 103.60  (15.28) | 108.95  (12.82) | <.001 | 103.97  (15.21) | 109.14  (12.29) | .008 | 101.81  (15.92) | 106.69  (13.32) | .012 | 105.54  (14.35) | 111.81  (12.50) | .005 |
| IQ-m | N (% male) | 194 (71%) | 171 (69%) | .8 | 64 (64%) | 62 (74%) | .3 | 68 (78%) | 64 (66%) | .2 | 62 (69%) | 45 (67%) | .9 |
|  | Age (yrs) | 15.74 (5.91) | 16.17 (5.94) | .6 | 22.71 (3.60) | 23.28 (3.10) | .3 | 14.88 (1.73) | 15.29 (1.76) | .3 | 9.47 (1.46) | 9.54 (1.57) | .8 |
|  | IQ | 108.49 (9.31) | 109.83 (8.99) | .2 | 109.73 (9.71) | 111.96 (9.70) | .3 | 106.76 (9.36) | 107.53 (8.99) | .6 | 109.11 (9.22) | 110.17 (7.25) | .3 |
