# Supplementary material for: Modeling flexible behavior in childhood to adulthood shows age-dependent learning mechanisms and less optimal learning in autism in each age group
Source: PLoS Biol. 2020 Oct 27;18(10):e3000908. doi: 10.1371/journal.pbio.3000908 (PMC7591042; doi:10.1371/journal.pbio.3000908)
Supplement: S5 Table — (DOCX) [file pbio.3000908.s017.docx]

|  |  | n | |  | % | | *χ*^2^ | p value |
| --- | --- | --- | --- | --- | --- | --- | --- | --- |
|  |  | Pass | Fail | Total | Pass | Fail |  |  |
| Children | TD | 31 | 33 | 64 | 48 | 52 | 0.0012 | 0.9724 |
|  | ASD | 39 | 42 | 81 | 48 | 52 |  |  |
| Adolescents | TD | 64 | 26 | 90 | 71 | 29 | 8.5657 | 0.00426 |
|  | ASD | 58 | 56 | 114 | 51 | 49 |  |  |
| Adults | TD | 81 | 16 | 97 | 84 | 16 | 5.5759 | 0.01821 |
|  | ASD | 88 | 38 | 126 | 70 | 30 |  |  |
| Total | TD | 176 | 75 | 251 | 70 | 30 | 9.4343 | 0.00213 |
|  | ASD | 185 | 136 | 321 | 58 | 42 |  |  |
| Total | Children | 70 | 75 | 145 | 48 | 52 | 30.051 | 2.981×10^−7^ |
|  | Adolescents | 122 | 82 | 204 | 60 | 40 |  |  |
|  | Adults | 169 | 54 | 223 | 76 | 24 |  |  |
