# Supplementary material for: Modeling flexible behavior in childhood to adulthood shows age-dependent learning mechanisms and less optimal learning in autism in each age group
Source: PLoS Biol. 2020 Oct 27;18(10):e3000908. doi: 10.1371/journal.pbio.3000908 (PMC7591042; doi:10.1371/journal.pbio.3000908)
Supplement: S6 Table — (DOCX) [file pbio.3000908.s018.docx]

|  |  | Children | Adolescents | Adults |
| --- | --- | --- | --- | --- |
| TD | EWA | 0.01 | 0.02 | 0.79 |
|  | RP | 0.14 | 0.94 | 0.18 |
|  | CU | 0.85 | 0.04 | 0.03 |
|  |  |  |  |  |
| ASD | EWA | 0.03 | 0.04 | 0.64 |
|  | RP | 0.12 | 0.78 | 0.11 |
|  | CU | 0.84 | 0.16 | 0.24 |
