# Supplementary material for: Modeling flexible behavior in childhood to adulthood shows age-dependent learning mechanisms and less optimal learning in autism in each age group
Source: PLoS Biol. 2020 Oct 27;18(10):e3000908. doi: 10.1371/journal.pbio.3000908 (PMC7591042; doi:10.1371/journal.pbio.3000908)
Supplement: S7 Table — (DOCX) [file pbio.3000908.s019.docx]

|  |  | m.weights |
| --- | --- | --- |
| TD | EWA | 0.000 |
|  | RP | **0.989** |
|  | CU | 0.011 |
|  |  |  |
| ASD | EWA | 0.004 |
|  | RP | **0.820** |
|  | CU | 0.176 |

m.weights = model weights using Bayesian model average with Bayesian Bootstrap
