# Supplementary material for: Modeling flexible behavior in childhood to adulthood shows age-dependent learning mechanisms and less optimal learning in autism in each age group
Source: PLoS Biol. 2020 Oct 27;18(10):e3000908. doi: 10.1371/journal.pbio.3000908 (PMC7591042; doi:10.1371/journal.pbio.3000908)
Supplement: S8 Table — (DOCX) [file pbio.3000908.s020.docx]

|  |  | | Children | |  | |  | | Adolescents | |  | |  | | Adults | |  | |
| --- | --- | --- | --- | --- | --- | --- | --- | --- | --- | --- | --- | --- | --- | --- | --- | --- | --- | --- |
|  | Win-stay | | Lose-shift | | PerErrors | | Win-stay | | Lose-shift | | PerErrors | | Win-stay | | Lose-shift | | PerErrors | |
|  | ASD | TD | ASD | TD | ASD | TD | ASD | TD | ASD | TD | ASD | TD | ASD | TD | ASD | TD | ASD | TD |
| *η* | 0.26* | 0.44*** | **0.66***** | **0.28*** | -0.24* | -0.55*** |  |  |  |  |  |  |  |  |  |  |  |  |
| *η*^rew^ |  |  |  |  |  |  | **0.49***** | **0.67***** | **-0.04** | **-0.33***** | -0.57*** | -0.61*** |  |  |  |  |  |  |
| *η*^pun^ |  |  |  |  |  |  | 0.29*** | 0.31*** | 0.14 | 0.19 | -0.21* | -0.42*** |  |  |  |  |  |  |
| *φ* |  |  |  |  |  |  |  |  |  |  |  |  | 0.18*** | 0.20 | -0.62 | -0.59 | 0.01 | -0.23* |
| *β* | 0.86*** | 0.87*** | **-0.21** | **-0.54***** | -0.34*** | -0.47*** | 0.86*** | 0.81*** | -0.52*** | -0.81*** | -0.29*** | -0.37*** | 0.87*** | 0.90*** | -0.61*** | -0.66*** | -0.41*** | -0.56*** |
| *α* | 0.14 | 0.04 | **0.27** | **-0.19** | -0.09 | 0.15 | 0.03 | 0.16 | 0.18 | -0.03 | -0.14 | -0.26* | 0.19* | -0.01 | -0.06 | -0.04 | -0.08 | 0.01 |
| *ρ* |  |  |  |  |  |  |  |  |  |  |  |  | -0.75*** | -0.84 | **0.29***** | **0.64***** | 0.80*** | 0.77*** |

PerErrors = Perseverative errors; * p < .05, ** p < .01, ***p < .005

**In bold, correlations that are significantly different from one another**
