# Supplementary material for: Modeling flexible behavior in childhood to adulthood shows age-dependent learning mechanisms and less optimal learning in autism in each age group
Source: PLoS Biol. 2020 Oct 27;18(10):e3000908. doi: 10.1371/journal.pbio.3000908 (PMC7591042; doi:10.1371/journal.pbio.3000908)
Supplement: S9 Table — (DOCX) [file pbio.3000908.s021.docx]

|  | | | Win-stay | Lose-shift | PerErrors |
| --- | --- | --- | --- | --- | --- |
| Age | Children | ASD | 0.01 | -0.06 | -0.06 |
|  |  | TD | 0.21 | -0.08 | -0.23 |
|  | Adolescents | ASD | 0.22* | -0.27*** | 0.02 |
|  |  | TD | 0.31*** | -0.45**** | -0.19 |
|  | Adults | ASD | 0.03 | 0.02 | -0.01 |
|  |  | TD | 0.15 | -0.07 | -0.005 |
| IQ | Children | ASD | 0.23* | -0.12 | -0.23* |
|  |  | TD | 0.13 | -0.05 | -0.22 |
|  | Adolescents | ASD | 0.42**** | -0.24** | -0.37**** |
|  |  | TD | 0.02 | 0.08 | -0.21* |
|  | Adults | ASD | 0.40**** | -0.26**** | -0.38**** |
|  |  | TD | 0.27** | -0.20 | -0.28** |
| *ASD only* | | | | | |
| ADI-R RRB | Children | | 0.07 | -0.23* | -0.14 |
|  | Adolescents | | 0.20* | 0.01 | -0.13 |
|  | Adults | | -0.31**** | 0.19* | 0.29**** |
| RBS-R Stereotyped | Children | | 0.12 | -0.06 | 0.006 |
|  | Adolescents | | 0.11 | 0.14 | -0.17 |
|  | Adults | | -0.18 | 0.24* | 0.04 |
| RBS-R Ritualistic- Sameness | Children | | -0.05 | -0.17 | 0.06 |
|  | Adolescents | | 0.09 | 0.18 | -0.21** |
|  | Adults | | -0.30**** | 0.23* | 0.18 |
| ADI-R Social Interaction | Children | | -0.14 | -0.005 | 0.02 |
|  | Adolescents | | 0.00003 | -0.02 | -0.10 |
|  | Adults | | -0.02 | 0.11 | 0.15 |
| ADI-R Communication | Children | | -0.17 | -0.06 | -0.04 |
|  | Adolescents | | 0.04 | 0.09 | -0.06 |
|  | Adults | | -0.10 | 0.14 | 0.21* |
| SRS-2 SCI | Children | | 0.11 | -0.06 | -0.05 |
|  | Adolescents | | 0.03 | 0.06 | 0.02 |
|  | Adults | | -0.05 | 0.19 | 0.12 |
| ADHD Hyperactivity/  Impulsivity | Children | | 0.07 | 0.04 | 0.14 |
|  | Adolescents | | -0.16 | 0.15 | 0.01 |
|  | Adults – parent-report | | -0.25* | 0.27** | 0.32**** |
|  | Adults – self-report | | -0.21* | 0.17 | 0.15 |
| ADHD Inattention | Children | | 0.07 | -0.06 | 0.15 |
|  | Adolescents | | -0.01 | -0.09 | 0.04 |
|  | Adults – parent-report | | -0.21* | 0.27** | 0.25* |
|  | Adults – self-report | | -0.01 | 0.04 | 0.11 |
| Anxiety (BAI/BYI-II) | Children | | -0.09 | -0.05 | 0.34*** |
|  | Adolescents | | -0.05 | 0.001 | 0.05 |
|  | Adults | | 0.14 | -0.09 | -0.14 |

ADI-R = Autism Diagnostic Interview-Revised; RBS-R = Repetitive Behaviour Scale-Revised; SRS-2 SCI = Social Responsiveness Scale 2^nd^ Edition Social Communication Index; BAI: Beck Anxiety Inventory; BYI-II: Beck Youth Inventories – Second Edition; PerErrors = perseverative errors; prop = proportion

PerErrors = Perseverative errors; * *p* < 0.05, ** *p* < 0.01, ****p* < 0.0045 (Children/Adolescent Bonferroni threshold – corrected *p* value = 0.05/11 = 0.0045), **** *p* < 0.0038 (Adult Bonferroni threshold – corrected *p* value = 0.05/13 = 0.0038)
