# Supplementary material for: Modeling flexible behavior in childhood to adulthood shows age-dependent learning mechanisms and less optimal learning in autism in each age group
Source: PLoS Biol. 2020 Oct 27;18(10):e3000908. doi: 10.1371/journal.pbio.3000908 (PMC7591042; doi:10.1371/journal.pbio.3000908)
Supplement: S10 Table — (DOCX) [file pbio.3000908.s022.docx]

|  | | | *η* | *η*^rew^ | *η*^pun^ | *φ* | *β* | *α* | *ρ* |
| --- | --- | --- | --- | --- | --- | --- | --- | --- | --- |
| Age | Children | ASD | -0.15 |  |  |  | 0.08 | 0.04 |  |
|  |  | TD | -0.03 |  |  |  | 0.25 | -0.18 |  |
|  | Adolescents | ASD |  | 0.06 | 0.06 |  | 0.23 | 0.03 |  |
|  |  | TD |  | 0.17 | -0.09 |  | 0.35*** | 0.05 |  |
|  | Adults | ASD |  |  |  | 0.05 | -0.02 | -0.02 | 0.04 |
|  |  | TD |  |  |  | -0.06 | 0.13 | -0.04 | -0.08 |
| IQ | Children | ASD | -0.02 |  |  |  | 0.27** | -0.03 |  |
|  |  | TD | 0.1 |  |  |  | 0.18 | 0.18 |  |
|  | Adolescents | ASD |  | 0.23 | 0.1 |  | 0.33*** | -0.07 |  |
|  |  | TD |  | 0.15 | 0.01 |  | -0.07 | 0.17 |  |
|  | Adults | ASD |  |  |  | 0.15 | 0.34**** | 0.11 | -0.33**** |
|  |  | TD |  |  |  | 0.15 | 0.25** | -0.04 | -0.27** |
| *ASD only* | | | | | | | | | |
| ADI-R RRB | Children | | -0.15 |  |  |  | 0.24 | 0.09 |  |
|  | Adolescents | |  | 0.15 | 0.1 |  | 0.12 | 0.06 |  |
|  | Adults | |  |  |  | -0.03 | -0.29**** | 0.04 | 0.24**** |
| RBS-R Stereotyped | Children | | 0.14 |  |  |  | 0.11 | -0.04 |  |
|  | Adolescents | |  | 0.05 | 0.05 |  | 0.06 | -0.001 |  |
|  | Adults | |  |  |  | -0.16 | -0.28** | -0.10 | 0.09 |
| RBS-R Ritualistic- Sameness | Children | | -0.09 |  |  |  | -0.11 | -0.08 |  |
|  | Adolescents | |  | 0.11 | 0.08 |  | 0.10 | 0.11 |  |
|  | Adults | |  |  |  | -0.09 | -0.32**** | -0.04 | 0.18 |
| ADI-R | Children | | -0.03 |  |  |  | -0.09 | -0.1 |  |
| Communication | Adolescents | |  | 0.09 | 0.07 |  | -0.05 | 0.1 |  |
|  | Adults | |  |  |  | -0.19 | -0.02 | 0.05 | 0.16 |
| ADI-R Social | Children | | 0.06 |  |  |  | -0.06 | 0.05 |  |
| Interaction | Adolescents | |  | 0.14 | 0.1 |  | -0.07 | 0.11 |  |
|  | Adults | |  |  |  | -0.12 | 0 | 0.05 | 0.1 |
| SRS-2 SCI | Children | | 0.03 |  |  |  | 0.24 | -0.14 |  |
|  | Adolescents | |  | 0.06 | 0.09 |  | 0.07 | -0.09 |  |
|  | Adults | |  |  |  | -0.18 | -0.18 | -0.04 | -0.07 |
| ADHD Hyperactivity/ Impulsivity | Children | | 0.01 |  |  |  | -0.01 | -0.22 |  |
|  | Adolescents | |  | -0.07 | -0.02 |  | 0.17 | -0.02 |  |
|  | Adults – parent-report | |  |  |  | -0.08 | -0.37**** | 0.07 | 0.1 |
|  | Adults – self-report | |  |  |  | -0.1 | -0.15 | 0.12 | 0.23** |
| ADHD Inattention | Children | | 0 |  |  |  | 0.05 | -0.08 |  |
|  | Adolescents | |  | -0.05 | -0.13 |  | 0.01 | -0.08 |  |
|  | Adults – parent-report | |  |  |  | -0.05 | -0.30**** | 0.05 | -0.03 |
|  | Adults – self-report | |  |  |  | -0.04 | 0.02 | 0.12 | 0.01 |
| Anxiety | Children | | -0.11 |  |  |  | -0.04 | -0.17 |  |
| (BAI/BYI-II) | Adolescents | |  | -0.12 | -0.06 |  | -0.1 | 0.17 |  |
|  | Adults | |  |  |  | 0.04 | 0.16 | 0.12 | -0.06 |

ADI-R = Autism Diagnostic Interview-Revised; RBS-R = Repetitive Behaviour Scale-Revised; SRS-2 SCI = Social Responsiveness Scale 2^nd^ Edition Social Communication Index; BAI: Beck Anxiety Inventory; BYI-II: Beck Youth Inventories – Second Edition

**p < .01, but doesn’t survive multiple comparison corrections, ****p* < 0.0045 (Children/Adolescent Bonferroni threshold – corrected *p* value = 0.05/11 = 0.0045), **** *p* < 0.0038 (Adult Bonferroni threshold – corrected *p* value = 0.05/13 = 0.0038)
