# Supplementary material for: Modeling flexible behavior in childhood to adulthood shows age-dependent learning mechanisms and less optimal learning in autism in each age group
Source: PLoS Biol. 2020 Oct 27;18(10):e3000908. doi: 10.1371/journal.pbio.3000908 (PMC7591042; doi:10.1371/journal.pbio.3000908)
Supplement: Attachment — Submitted filename: PRL_Response to reviewers comments_DC_LZ.pdf [file pbio.3000908.s023.pdf]

30thth May 2020

Manuscript ID: PBIOLOGY-D-19-03505R1

Manuscript Title: “Modeling flexible behavior in children, adolescents and adults with autism spectrum disorder and typical development”

Dear Dr Gasque,

Thank you for providing us with an opportunity to respond to the points raised by the reviewers. We would also like to thank the reviewers for their time and appreciate their helpful comments and suggestions. We have addressed all points raised by the reviewers below, and in our revised submission. We believe that the revisions have resulted in a stronger, more balanced manuscript.

Reviewers comments are in **bold**, our replies are in blue standard font and, where applicable, we include in *blue italics* text we have made substantial changes to in the manuscript or supporting information (and note their location). All changes in the manuscript have been specified in the edited, tracked changes version. Additionally, we have provided one clean version. Locations of edits described below align with the clean version.

We hope that the manuscript may now be acceptable for publication in PLoS Biology.

Yours sincerely,
Daisy Crawley and Lei Zhang (on behalf of the co-authors)

Reviewers' comments and responses

Reviewer #1:

1. It is interesting that the authors choose to use a probabilistic reversal learning (PRL) task to study flexibility in autism, rather than a traditional cognitive flexibility task. This is because the PRL task includes a reward outcome. Thus, both flexibility and reward processing are indexed by such a task, and reward processing may also be altered in ASD. Can the authors elaborate in the Introduction on their decision to use such a task, rather than some variant of the Wisconsin Card Sort Task or other task-switching or set-shifting paradigms that do not involve a reward component?

Response 1: We appreciate the reviewer's interest in our choice of task and thank them for suggesting we elaborate more on the task decision. We have added the following explanation in the Introduction (see Line 141):

“Probabilistic reversal learning paradigms provide a direct assessment of flexible choice behavior (in addition to tapping reinforcement learning), as they require

information to be integrated over a number of trials in order to detect true changes and – much like interacting with our environment – this trial-and-error learning is continually updated throughout the task. Furthermore, PRL paradigms do not require tracking of extra-dimensional shifts, thereby constraining the recruitment of additional cognitive domains (Nilsson et al., 2015, Schmitt et al., 2019).”

With respect to the reward component, we highlight that most task-switching paradigms, including the Wisconsin Card Sort Task, also involve using feedback to guide choice behavior.

2. Is there previous literature to indicate that the specific reward/punishment stimuli used in this task are appropriately sensitive for indexing reward/punishment in both ASD and TD groups across age?

Response 2: We thank the reviewer for this interesting query. Smiling and sad faces are commonly used reward/punishment stimuli, and are frequently paired with green and red coloring, respectively. These stimuli are consistent with previous studies of reward processing in both ASD and TD samples (Scott-Van Zeeland et al., 2010; den Ouden et al., 2013; Yerys et al., 2009). In addition, a bell chime was presented with reward stimuli and an “incorrect” buzzer sound was presented with punishment stimuli, which are relatively universally salient and appropriately sensitive. Information concerning the sound types has been added to the paradigm description for full clarity (Line 239):

“Positive feedback consisted of green, smiling emoticons and negative feedback of red, frowning emoticons (i.e. reward/punishment) and accompanying sounds (bell chime/buzzer, respectively).”

We have also added the following to the discussion to capture suggested future directions with respect to different stimuli types (Line 704):

“Additionally, given the growing literature suggesting differential reward processing in ASD, future work could assess potential differences in learning and flexible behavior in the context of different reward modalities i.e. use different types of feedback, such as monetary stimuli.”

3. Why was reaction time not assessed in addition to accuracy? It would be helpful to include this information as well as statistics comparing groups.

Response 3: Reaction time was not assessed in the current study (nor in fact in any of the other studies that use this task; Murphy *et al.*, 2003; Chamberlain *et al.*, 2006; den Ouden et al., 2013; Lawrence, Sahakian, Rogers, Hodge, & Robbins, 1999) as the task does not impose a time limit for responding nor instruct individuals to respond quickly and therefore reaction times are widely distributed and do not quantify flexible behavior. However, because of the reviewer question we briefly assessed reaction times, and based on these analyses we believe that including these reaction time results does not have added value.

First, we generated within-subject z-scored reaction times (z-RT) and examined their distribution both overall and within diagnosis and age groups (Figure R1A-B). It is clear that this RT distribution has a longer tail than the typical RT distribution, denoting a high variability of RTs on this task.

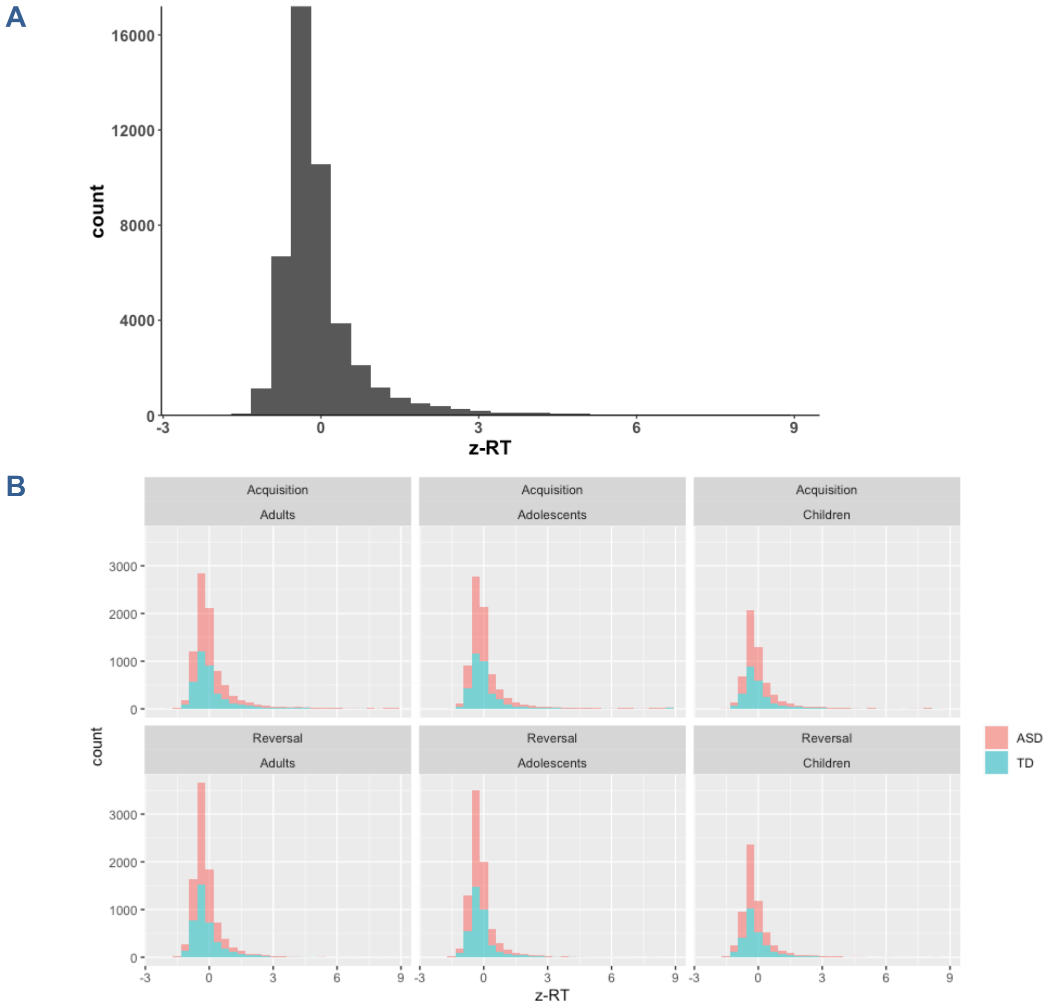

Figure R1: Histogram of z-scored reaction times (A) across the full sample, and (B) split by phase and age group; red = ASD, blue = TD

Additionally, Figure S1 (newly added, presented below) presents average, per trial, reaction times decrease over the course of the task, reflecting a general speeding up across trials, rather than increased task accuracy or flexible behavior (as participants are more accurate in the acquisition phase; see Figure 2B and Figure S3). Importantly, there is no evidence of a significant increase in reaction time at the point of reversal (trial 40), again illustrating that RTs are unlikely to reflect task-relevant processes.

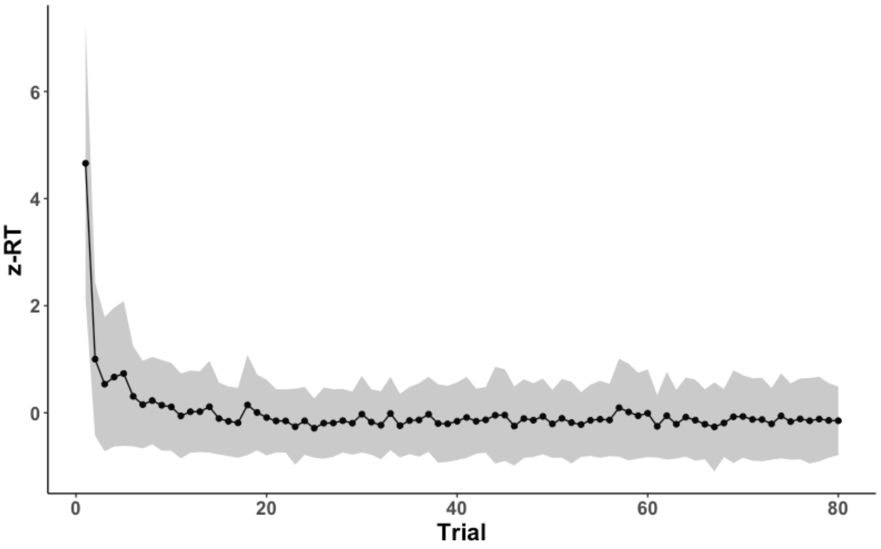

"Figure S1: *Reaction times (z-scored; z-RT) in the PRL task averaged across task trials; shaded area represents the standard deviation. Notably, reaction times do not change at the point following reversal, illustrating that reaction times are unlikely to reflect task-relevant processes."*

Finally, comparing diagnostic groups showed no significant difference between ASD and TD individuals on average (p=.7), demonstrating that reaction time is not driving group differences in flexible behavior.

To briefly reflect this discussion, we have added clarification to the manuscript regarding why reaction time is not an index of flexible behavior in this task (see Line 278, below) and added Figure S1 to the Supporting information:

"As in previous studies using this task (Chamberlain et al., 2006; Cools, Clark, Owen, & Robbins, 2002; den Ouden et al., 2013; Lawrence et al., 1999; Murphy, Michael, Robbins, & Sahakian, 2003), reaction time is not examined here because it is unlikely to capture task-relevant processes since no response speed instructions are given nor is there a time limit for responding (see Figure S1 for further discussion)."

4. It is very surprising that the authors do not attempt to match the ASD and TD groups on full-scale IQ. It would be best to take a subset of the larger sample that is matched on full-scale IQ to see if the results obtained from the larger sample still hold. It is not sufficient to simply include IQ as a confounding regressor.

Response 4: We thank the reviewer for this suggestion and acknowledge the continued debate regarding how to address IQ matching in ASD research (e.g. Jarrold & Brock, 2004). We agree with the reviewer that using IQ as a confound regressor can only capture linear effects of IQ. The reviewer's great suggestion to conduct analyses in a subset of individuals matched on full-scale IQ, addresses this issue. Using the 'MatchIt' package (Ho, Imai, King, & Stuart, 2011), we found that results are largely unchanged, with comparable means and variance in the data. We

present the results in the Supporting information with reference to the analyses in the manuscript (Line 428):

"Therefore, for all further group comparisons we assessed whether results changed with IQ as a confound regressor and, in addition, we conducted analyses of task behavior in an IQ-matched subsample (Text S2, Table S3). Results were largely unchanged throughout (see Text S2, Figure S2)."

The following is presented in the Supporting information (Text S2; Figure S2, Table S3):

"In addition to the above analyses examining IQ as a confound regressor, we conducted further post-hoc analyses with an IQ-matched subsample. Here, we used the "exact matching" method ('MatchIt' package), taking individuals from within an IQ range of 90-130 matching both the mean and variance. Table S3 presents IQ mean, standard deviation and range of the full sample and IQ-matched subsample, split by diagnostic group, overall and within each age group. Figure S2 presents within-age group between-diagnostic group comparisons on all four task behavioral measures (Figure S2E-H; analyses discussed below), with results from the full sample analyses presented above (Figure S2A-D) for reference. The pattern of results is largely unchanged.

Table S3: *Descriptive statistics (mean, SD – unless otherwise stated) for the full sample and the IQ-matched (IQ-m) subsample, within age and diagnostic groups, with p values for within-age group, between diagnostic group comparisons of age, sex and IQ*

		Total			Adults			Adolescents			Children		
		ASD	TD	p	ASD	TD	p	ASD	TD	p	ASD	TD	p
Full sample	N (% male)	321 (72%)	251 (68%)	.3	126 (70%)	97 (72%)	.8	114 (76%)	90 (69%)	.3	81 (70%)	64 (61%)	.3
	Age (yrs)	16.67 (5.92)	16.93 (6.02)	.6	22.80 (3.55)	23.25 (3.29)	.2	14.94 (1.71)	15.39 (1.71)	.1	9.59 (1.50)	9.52 (1.54)	.7
	IQ	103.60 (15.28)	108.95 (12.82)	<.001	103.97 (15.21)	109.14 (12.29)	.008	101.81 (15.92)	106.69 (13.32)	.012	105.54 (14.35)	111.81 (12.50)	.005
IQ-m	N (% male)	194 (71%)	171 (69%)	.8	64 (64%)	62 (74%)	.3	68 (78%)	64 (66%)	.2	62 (69%)	45 (67%)	.9
	Age (yrs)	15.74 (5.91)	16.17 (5.94)	.6	22.71 (3.60)	23.28 (3.10)	.3	14.88 (1.73)	15.29 (1.76)	.3	9.47 (1.46)	9.54 (1.57)	.8
	IQ	108.49 (9.31)	109.83 (8.99)	.2	109.73 (9.71)	111.96 (9.70)	.3	106.76 (9.36)	107.53 (8.99)	.6	109.11 (9.22)	110.17 (7.25)	.3

Task behavioral analyses were then re-run using the IQ-matched subsample. As shown in Table S2, diagnostic groups did not differ on sex, age or IQ, either overall or within each age group (ps>.2). A repeated-measures analysis of accuracy showed significant main effects of phase ($F_{(1,359)}$=199.99, p<2.2×10^{-16}), diagnosis ($F_{(1,359)}$=12.40, p=.0005) and age group ($F_{(2,359)}$=21.53, p=1.06×10^{-8}), but no significant interactions (all ps>.3), as in the full sample. Post-hoc analyses confirmed the same pattern as in the full sample analyses – accuracy was on average significantly higher: (i) in the acquisition phase than the reversal phase (M_{acq}=0.79, SD_{acq}=0.15, M_{rev}=0.66, SD_{rev}=0.19), (ii) in TD individuals compared to ASD individuals (M_{TD}=0.75, SD_{TD}=0.17, M_{ASD}=0.69, SD_{ASD}=0.18) and (iii) in older age

groups compared to younger age groups (Adults-Adolescents, p=.015, 0; Adults-Children, p<.0001, d=0.80; Adolescents-Children, p=.0002, d=0.42).
Next, the significant main effect of diagnosis on perseverative errors was also present in this subsample ($F_{(1,357.66)}$=4.27, p=.023, d=0.26), such that ASD individuals made on average significantly more perseverative errors than TD individuals. As in the full sample, there was no significant effect of age nor interaction between diagnosis and age group (ps>.08).

Feedback sensitivity analyses also replicated the full sample findings: ASD individuals again showed on average significantly less win-stay and more lose-shift behavior relative to TD individuals, and for both there was a main effect of age (win-stay: diagnosis ($F_{(1,357.37)}$=7.22, p=.0075, d=0.24), age group ($F_{(2,303.82)}$=30.48, p=8.64×10^{-13}); lose-shift: diagnosis ($F_{(1,359)}$=8.38, p=.0004, d=0.37), age group ($F_{(2,359)}$=14.89, p=6.16×10^{-7}). Pairwise post-hoc comparisons revealed win-staying increased and lose-shifting decreased with age.

Finally, for win-stay behavior, the predicted interaction between diagnosis and age group was not significant in this subsample (p=.2), however, the pre-planned within age-group between-diagnosis group analysis did show, as in the full sample, that ASD adolescents displayed less win-staying than TD adolescents as in the full sample analyses (p=0.0050, d=0.51). This analysis survived Bonferroni correction (correcting for task behavioral measures × age groups: p value =.05/(3×3)=.0056), as in the full sample analyses. For lose-shift behavior, there was no significant interaction between diagnosis and age group (p=.3) and no between-diagnosis group age group comparisons survived Bonferroni correction (ps>.015), replicating the patterns of results in the full sample findings.

In summary, analyses on an IQ-matched subsample are consistent with the full sample findings, which is in line with the observation that inclusion of IQ as a confound regressor in the full sample did not affect the pattern of results (see Figure S2)."

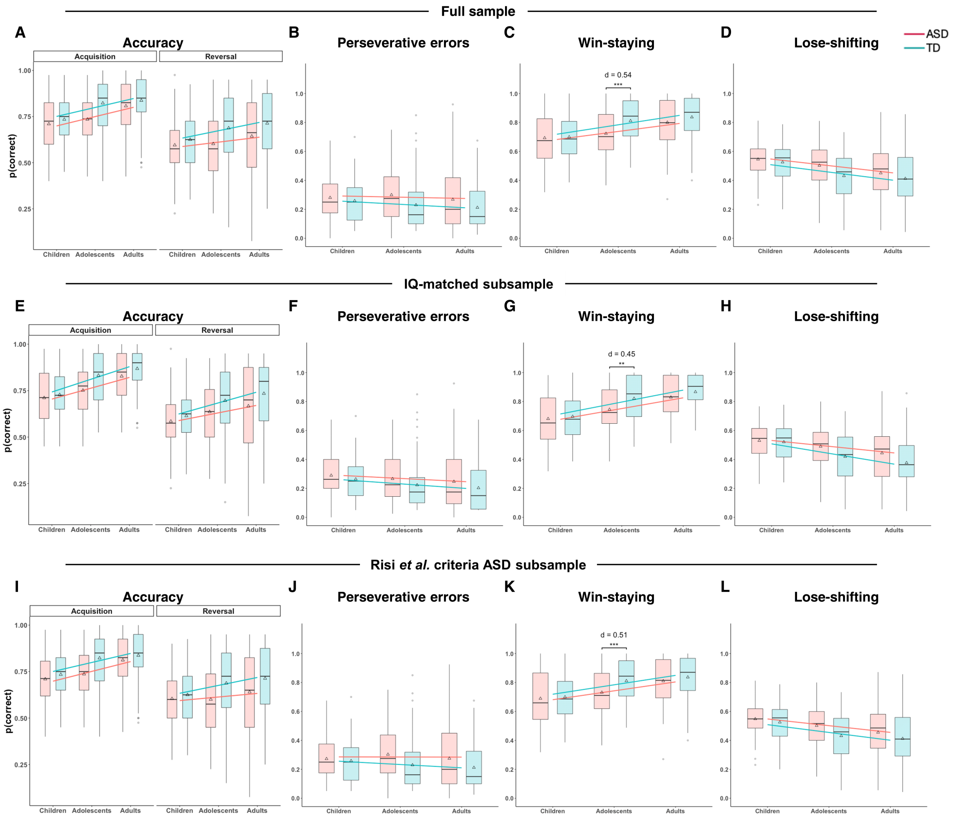

*“**Figure S2:** Boxplots showing task behavior for (A-D) the full sample, (E-H) the IQ-matched subsample and (I-L) the Risi et al. ADI-R criteria ASD subsample**.** The pattern of results remains largely unchanged across both subsample analyses.”*

5. The authors may wish to see the following relevant work:

The paradox of cognitive flexibility in autism. Geurts HM, Corbett B, Solomon M. Trends Cogn Sci. 2009 Feb;13(2):74-82.
Demystifying cognitive flexibility: Implications for clinical and developmental neuroscience. Dajani DR, Uddin LQ. Trends Neurosci. 2015 Sep;38(9):571-8.

Response 5: We thank the reviewer for highlighting these papers, to which we now refer (Lines 132,154).

Reviewer #2:

FP model specification
1. One of the models is labelled ‘fictitious play’ (FP) and consists in a Rescorla-Wagner model where the value of the unchosen option is also updated (with the opposite prediction error). The first (but not the main) issue with this model is that the name is wrong and misleading. In behavioural game theory, ‘fictitious play’ refers to a way of finding a best response to an opponent play by iteratively mentally simulating the other player response: a situation that clearly does not apply here, as FP learning concerns beliefs, not values.

Response 1: Thank you for this comment. We see the concern of the reviewer and have now changed "fictitious update" to "counterfactual update" throughout the manuscript to avoid further confusion.

The more important issue concerning this model is that, in its current specification, the factual and counterfactual updates are governed by the same learning rate. This is problematic as the functional complexity (it has an additional equation compared to the Rescorla-Wagner) of the model is not quantified by an additional parameter. This is also problematic as it does not allow to quantify the counterfactual update separately from the factual one. It would be psychologically plausible to suppose that the two are differentially affected by ASD and age.

Regarding model complexity, we would like to point out that our method of model comparison does indeed account for this increased functional complexity in the counterfactual update model, unlike simpler methods based on e.g. AIC/BIC comparison. This is one of the motivations from our perspective to conduct all computational modeling analyses in the Bayesian framework, where inferences are drawn from joint posterior distributions, rather than point estimates. Under the Bayesian framework, the joint parameter spaces of the CU and the simple RL model differ; thus, the effective number of parameters of these two models is different. It is the latter (i.e., effective number of parameters) that we use in the penalizing term in computing the model evidence.

In response to this interesting discussion, the following has been added to the supporting information (Text S1; Figure S7):

"Whether it is necessary to separately quantify learning rates for the counterfactual update is an empirical question that can be answered using model comparison. When performing this model comparison, the model with a single learning rate was favored (ΔLOOIC > 100). In line with this, the estimated learning rates from the dual learning rate model are highly correlated (r > 0.95, see Figure S6), and the posterior distributions of the two learning rates largely overlap (95% HDI of the difference credibly contains 0), suggesting that indeed counterfactual learning occurs at the same rate as the 'normal' learning."

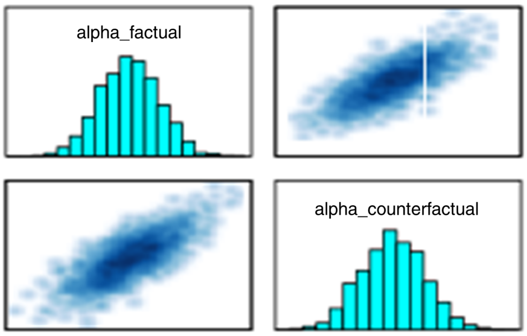

***"Figure S7.** Highly correlated factual and counterfactual learning rates."*

EWA model specification

2. Another model is the one labelled 'Experience-Weighted Attraction' model. I have also an issue concerning this labelling. Again, the EWA model has been developed for game theory not bandits; as this paper is addressed to psychologists and neuroscientists, rather than game theorists, I find the labelling unfortunate. Furthermore the key distinctive feature of the EWA model is counterfactual update (i.e., update of the value of the unchosen strategy, based on the opponent's choice), which is not relevant here. If anything the current model is closer to Erev and Roth's model (American Economic Review, 1995) than the EWA.

Response 2: Thank you for the comment. Indeed, we are aware that the EWA model is commonly used in strategic games. The application of this model to the current PRL task was first reported by den Ouden et al. (2013), and was labeled EWA there, and consistency across our studies is the first reason to keep this label. As we discussed in den Ouden et al. (2013), the EWA model used is a reduced version of the full EWA model proposed by Camerer & Hua Ho (1999). Our reason in the 2013 paper to retain the name is that the feature that gives the EWA model its name, i.e. experience-weighted attraction, is still retained in this reduced model (through the 'rho' parameter), and therefore we consider the name still applicable. To avoid confusion, we have now highlighted the reduced nature of this model version, replicating the model as specified in den Ouden et al. (2013) (Line 324):

"We examined this mechanism using the experience weight parameter from a reduced version of the experience-weighted attraction model as presented in previous work (EWA; den Ouden et al., 2013; for the full model, see: Camerer & Hua Ho, 1999). The original model has additional features relevant to multiplayer game modeling that are not applicable to the current PRL task paradigm and thus are not included here (in line with den Ouden et al., 2013)."

Yet, labeling is not the main issue I have with this model. In this paper the EWA model embodies an interesting (and plausible) idea, that is, when "experience" increases (i.e., number of trials per option) new outcomes count less. The problem is that it does so in a formalism that is quite different compared to that used in the other two models. For instance, while for some sets of parameter the EWA model can approximate the RW model, for many other parameters values option values will converge to very different quantities. Furthermore, the fact that experience weights can increase unboundedly (when the number of trials increases), is at odds with neurobiologically plausible instantiations of RL (see any basal ganglia model, for instance). To obviate this issues and ensure commensurability with the other models, the authors should replace the EWA model with Miller's model (Psychol Rev. 2019), which suppose a parallel 'habit' learning (equivalent to the experience weight) with a formalise that is closer to that of the other models and parameters that are psychologically easy to interpret.

We disagree with the reviewer that the EWA model and RL models are substantially different. We will reply to specific comments from the reviewer to support this view:

"For some sets of parameter the EWA model can approximate the RW model"
Indeed, in fact as noted in den Ouden et al. (2013), for rho = 0, $n_{C,t}$ is always 1, and the model is mathematically equivalent to the Rescorla-Wagner model.

"For many other parameters values option values will converge to very different quantities."
Using simulation (see below Fig. R4), we observe that the value curve and choice probability curve are qualitatively similar to RL models.

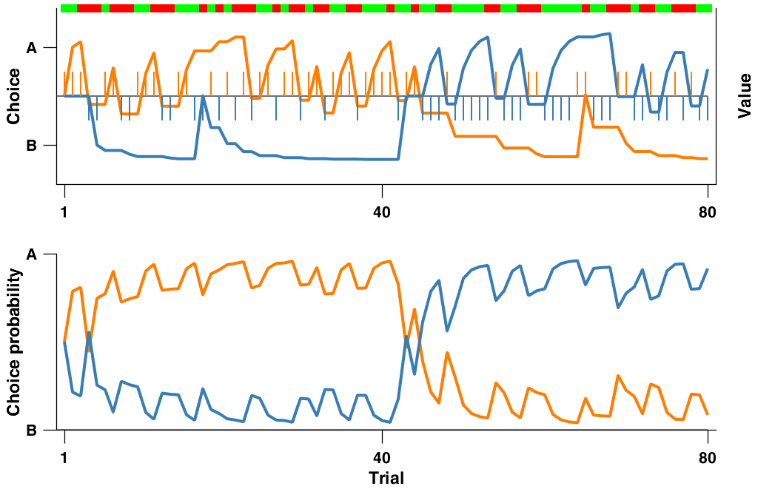

Figure R4. EWA model. Top Panel: sticks represent actual choices, curves represent choice values, and colors at the top denote actual outcome (green and red for positive and negative feedback, respectively). Bottom panel: choice probability computed from the model.

"The fact that experience weights can increase unboundedly (when the number of trials increases), is at odds with neurobiologically plausible instantiations of RL (see any basal ganglia model, for instance)."
We agree that if the experience weight could increase unboundedly, this would be biologically implausible. However, this is not the case. The experience weight n_t asymptotes to 1/(1-rho). At asymptote, $n_t = n_{t-1}$. Let's then replace $n_t = n_{t-1}$ = x and solve:

$$x=x*rho+1$$
$$x-x*rho = 1$$
$$x(1-rho)=1$$
$$x = 1/(1-rho)$$

This of course holds for when 0<rho<1. As we pointed out in our 2013 paper, for the boundary cases, when rho=0, this model reduces to the mathematical equivalent of simple Rescorla-Wagner, while for rho=1, the learnt value is a weighted average of all rewards, which is conceptually very similar to a standard Bayesian learner. Like a Bayesian learner, at this rho value, the learned values become impervious to new information as the prior belief (i.e. value) becomes increasingly narrow with increasing trial numbers.

Furthermore, as discussed above, we aim to keep the current study as close as possible to den Ouden et al. (2013). We indeed replicate the finding in adults that this EWA model is superior to an RP model. If we omit this model in the current manuscript, future readers will find it difficult to establish the link between our current and previous work. We now have made this argument clearer when introducing the EWA model (Line 345):

"Previous work has shown the EWA model to be the winning model in neurotypical adults in the same PRL task (den Ouden et al., 2013)."

Finally, with regard to the arbitration model (Miller et al., 2019), we thank the reviewer for this suggestion, but we argue that this model is not suitable for the current task design. The key feature of the model proposed in Miller et al. (2019) is that it can dissociate between habitual and goal-directed drivers of behavior, such as in the 2-step task. However, in our very simple reversal task with on every trial a choice between the same 2 stimuli, it is not possible to dissociate these controllers.

Softmax specification

3. The authors included a bias term in the softmax. I have issue with this parameter as I suspect it is capturing part of the effects induced by less ad hoc processes such as learning asymmetric, counterfactual update and increased habits. I suspect its value is consistent with a bias toward the first rewarded stimulus. Am I correct? In any case, I would not include it, unless it is strongly justified by model comparison and model falsification.

Response 3: We thank the reviewer for raising the concerns regarding the bias parameter in the softmax function. In fact, this bias term is often used as one additional parameter in the softmax choice rule (e.g., Gläscher et al., 2009; Lesage et al., 2017) – capturing the bias when the option values are indifferent. This bias does not necessarily reflect the first rewarded stimulus. In Table R2, we show that the posterior values of the bias parameter are both negative (bias towards the rewarded stimulus) and positive (bias towards the alternative). Moreover, in our current dataset, adding this bias term consistently improves every model's performance (see Table R2, below). We have now added a brief clarification (see Line 361):

"Including this indifference point parameter systematically improved performance of all models."

Table R2. Model comparison for models with and without indecision bias (α)

		Children		Adolescents		Adults	
		with α	without α	with α	without α	with α	without α
TD	EWA	5813	5845	6436	6481	6473	6553
	RP	5801	5813	6365	6429	6488	6580
	CU	5786	5830	6421	6504	6559	6647
ASD	EWA	7621	7714	10247	10382	9334	9442
	RP	7580	7720	10226	10350	9372	9469
	CU	7560	7682	10254	10467	9366	9581

Model evidence shows as leave-one-out information criteria (LOOIC), lower LOOIC value indicates better model fit. CU = counterfactual update model, RP = reward punishment model, EWA = experience weighted attraction model.

Model space specification
4. I think the model space should include the RW model + the full model that include all the features (counterfactual update learning rate; two learning rates for positive and negative prediction errors and the habit learning system of Miller) as two "extreme" benchmarks (hypo n vs. hyper-parameterisation).

Response 4: We thank the reviewer for suggesting the model space. We agree that ideally we would include a 'full' model such as suggested here. The reason we did not do this in the first place was that we worried that the complexity of this model was not warranted by the number of data points; this task only has 80 trials and a single reversal. Triggered by this comment, however, we decided to fit a model that combined counterfactual learning with pos/neg learning rates and EWA. This exercise confirmed our worries, as this model did not converge.

We now make clearer in the paper what we can and cannot infer from our model comparison results. Model comparison within the current model space cannot show that the processes captured by the non-winning models in each age group are not present in that age group at all. However, as explained in the manuscript (Line 817), they show that their relative dominance changes with age but, importantly, not with ASD diagnosis. This is where the true novelty of this paper lies: being the first to compare reinforcement learning processes in ASD and comparison controls across age groups.

Nonetheless, we appreciate these models and the combinations of these models, and we have now discussed the convergence issue and pointed out the possibility to further develop models as a future line of research in the Discussion section (Line 743):

"Secondly, it is important to note that each group's winning model is only relative to the other models tested here – although note that the models capture behavior well and perform far above chance. However, it is (always) possible that other models

may perform even better and further models may be developed in the future. A full model with all parameters combined was not possible due to convergence issues, emphasizing the relative dominance of learning mechanisms rather than any suggestions of mutual exclusivity. We highlight, nevertheless, that this study is the first to compare reinforcement learning models in ASD across age groups."

Model comparison and selection
5. I really liked the Bayesian model selection approach and the fact that authors showed also the simulation. However it would be interesting to see how the models perform on the other behavioral metrics (perseveration errors, win/stay etc.) to have a better idea of what behavioral feature is falsifying the "losing" models. It would also be important to show the model recovery on simulated datasets (i.e., the capacity to retrieve the correct model by model comparison in data where the ground truth is known). Finally, it would be interesting to know which is the "overall" winning model (across all ages).

Response 5: We thank the reviewer for the appreciation of our Bayesian model selection approach, and for encouraging us to examine the model performance on other behavioral metrics. We appreciate the usefulness of model falsification, but we argue that this is not the case in the current study. We believe that model falsification is particularly helpful when developing new models, but in the current study, all three models are well-established. In addition, a "losing" model in one group can be the "winning" model in another group. As we stated above, model falsification cannot show that the processes captured by the non-winning models in each age group are not present in that age group at all. However, they show that their relative dominance changes with age, but, importantly, not with ASD diagnosis.

As for the model recovery, as the basic Rescorla-Wagner model was always the worst, we ran a model recovery analysis using the three main models in our study (counterfactual RL, R-P, and EWA), with data from 85 simulated participants (averaged sample size across TD age groups). Results show that all models can be well recovered. The methodology is now included in the supporting information (Text S1, Line S109):

"Following establishment of the winning models, we conducted model recovery analyses using simulated data from 40 participants. The basic Rescorla-Wagner model was omitted as all other models consistently outperformed it. Results showed that all models' identities can be well recovered (Figure S4)."

The results are presented in Figure S4 and referenced in the manuscript (Line 502):

"Model recovery results showed that all models' identities can be well recovered (Figure S4)."

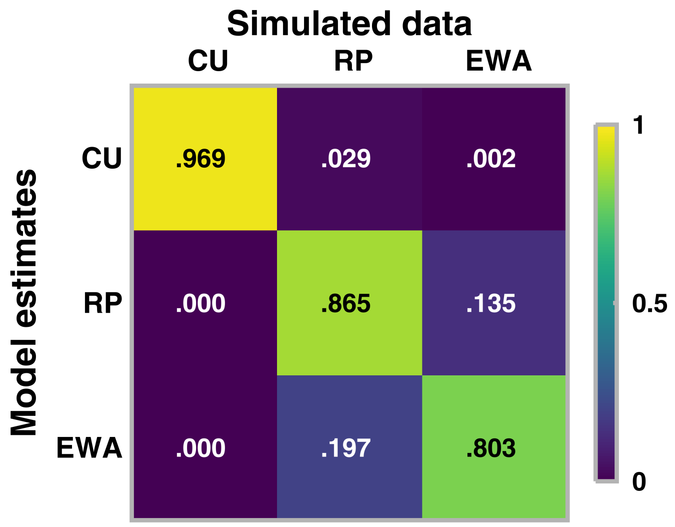

*“**Figure S4.** Model recovery. Data from 85 synthetic participants (averaged sample size across TD diagnostic group) were simulated with each of our three main models: CU = counterfactual update model, RP = reward punishment model, EWA = experience-weighted attraction model (note: we did not include the standard Rescorla-Wagner model as our three main models consistently performed better). Color indicates model weights calculated with Bayesian model averaging using Bayesian bootstrap (higher model weight value indicates higher probability of the candidate model to have generated the observed data).”*

As for the overall winning model, we indeed fit models to data from all age groups and we have provided these results in the supporting information (Table S6). Overall, the reward-punishment RL model is the winning model, regardless of TD or ASD. Results are now referenced in the manuscript (Line 503):

“Collapsing age groups, the R-P model provided the highest model evidence in both diagnostic groups (Table S6).”

Statistics on the parameters
6. I guess the parameters are correlated between them (to some extent, it is always the case). It would be interesting to verify the parameter recovery to check their correct estimability (similar to model recovery). Maybe a structural equation modeling (SEM)- approach would be useful to assess the effect of clinical score while controlling for the correlation between parameters and between scores. I also suspect that in the population, there are subjects whose behavior is random (i.e., the likelihood of any model is not different from assuming random choices). It would be interesting to check the correlations (or SEM results on the non-random subjects (as parameters values of random subjects do not make sense). Finally, once the "full" model implemented, it would be interesting to perform the correlations (or SEM) on the parameters of the full model.

Response 6: We thank the reviewer for suggesting we look at the correlations among parameters. However, as we used hierarchical Bayesian estimation and we implemented the non-centered reparameterization (to reduce the inter-dependency among parameters), parameters are not necessarily correlated. We examined the pair-wise association among the parameters, and we found that all parameters have independent contributions to the model (see Figure S4 below). We have added the following information to the manuscript (Line 495) and the below figure to the supporting information (Figure S4):

"Model weightings are shown in Figure 3A, and all winning model's parameters had independent contributions (Figure S4)."

Relatedly, given that we used hierarchical Bayesian estimation and examined parameters under the joint distribution principle (Gelman et al., 2013), when parameters are not correlated, it is warranted that parameters can be recovered. We are aware that parameter recovery is necessary when models are estimated using the more traditional maximum likelihood estimation, but it is somewhat redundant (though "good to have") when using the full Bayesian approach and when parameters are shown not to be correlated. And since the parameters are not correlated, it is not necessary to include the SEM analysis.

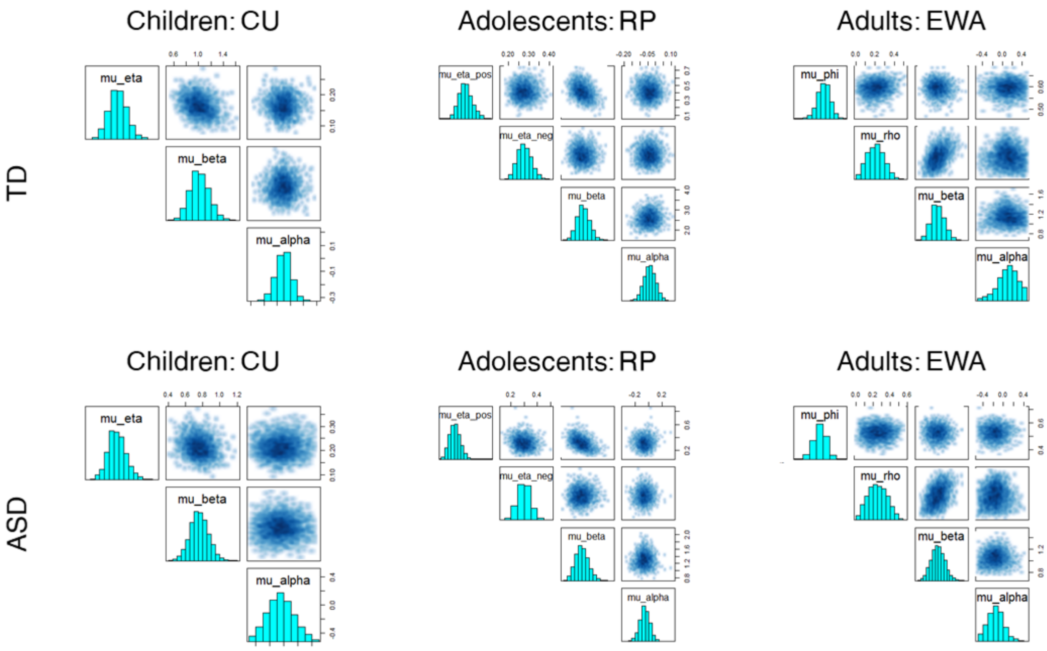

*"**Figure S4**. Independent contribution of model parameters. Pair plots of each group's winning model parameters for ASD (top panel) and TD (bottom panel). In each pair plot, diagonal plots show marginal distributions of each parameter, off-diagonal plots show pair-wise scatters of parameters. CU = counterfactual update model, RP = reward punishment model, EWA = experience-weighted attraction model."*

Discussion beyond ASD

7. I know that it is not central in this study, but, as I believe that authors should

mention that fact that that while some studies (we found it: Lefebvre et al 2019; others too: Van Slooten et al, 2018, 2019) showed higher learning rates for positive compared to negative learning rates, some others (Niv et al 2011; Gershman, 2015) found the opposite. As the present study contributes to this debate, it would be worth mentioning the "controversy" in the discussion.

Response 7: We thank the reviewer for encouraging us to contribute to this debate. We feel that the focus of the main manuscript discussion with respect to positive and negative learning (reward/punishment) is on the diagnostic group difference in reward learning in adolescents, however, we have added the following line to the supporting information to reflect these active discussions within the literature (Text S1, Line S128):

"These findings contribute to continued debates within the literature regarding higher learning rates for positive value compared to negative value stimuli (see also Lefebrvre et al., 2018; Van Slooten et al., 2018; Gershman, 2015)."

8. Line 157
Please avoid the term "poor" in describing adolescent decision-making

Response 8: We thank the reviewer for highlighting this wording, and have removed the word poor. The relevant sentence now reads (see Line 167):

"During adolescence, notable changes in goal-directed decision-making occur, often manifesting in risky decisions thought to be attributable to hypersensitivity to rewards."

Reviewer #3:

1) I was surprised not to see the ADOS (and only the ADI-R) - was the ADOS collected ? I did not see information allowing me to know whether all participants in the sample were above overall ADIR cut-off for an ASD, or above SRS cut-off. If not, have the authors tried to restrict their analysis to the subsample that meets all diagnostic criteria?

Response 1: We thank the reviewer for these observations and suggestions. ADOS scores were indeed collected for this sample, and below (Table R3) we present descriptive information for the total ASD sample and the three age groups, using calibrated severity scores (CSS) that allow for comparisons across ADOS modules (range 0-10; Gotham et al., 2009). However, we did not use ADOS data as indices of symptom severity as both the ADI-R and RBS-R/SRS-2 provide more dimensionality in assessing symptoms. Furthermore, ADOS scores of RRB are somewhat limited in their indication of RRB due to the short snapshot nature of observational measures such as the ADOS (Leekam, Prior, & Uljarevic, 2011). For this reason, the RBS-R and ADI-R were used to assess RRB, with the former being the most commonly used questionnaire measure of RRB and the latter providing a clinical capture of RRB that is superior to the ADOS.

Table R3. ADOS classifications and scores (mean, SD) for the ASD sample

ADOS	Total sample	Adults	Adolescents	Children
% of sample with ADOS CSS data	84%	80%	80%	95%
% with CSS scores in ASD range (>3)	65%	58%	74%	64%
Total mean (SD)	5.19 (2.73)	4.77 (2.63)	5.74 (2.78)	5.06 (2.72)
SA mean (SD)	5.91 (2.60)	5.61 (2.57)	6.42 (2.57)	5.71 (2.62)
RRB mean (SD)	4.72 (2.69)	4.65 (2.54)	4.82 (2.56)	4.70 (3.05)

CSS = Calibrated severity scores

With respect to comments regarding cut-offs, it has previously been shown that instrument cut-offs, even for gold-standard instruments, do not necessarily give better stability of diagnosis (C. Lord et al., 2006). Clinical judgment consistently augments diagnostic stability, beyond that of standardized instruments alone. Thus our criteria of a clinically diagnosed sample gives, we would argue, a more representative ASD sample that enables a better capture of the autism phenotype (Charman et al., 2017).

Nonetheless, we examined cut-offs following the reviewer's comments and have added the results to the Supporting information, with reference to these analyses in the manuscript:

Line 222 –
"Although ASD individuals were additionally assessed using the Autism Diagnostic Observation Schedule Catherine Lord et al., 2000; Catherine Lord et al., 2012 and Autism Diagnostic Interview-Revised (ADI-R, Rutter, Le Couteur, & Lord, 2003), reaching instrument cut-offs were not inclusion criteria as clinical judgment has been found to consistently improve diagnostic stability C. Lord et al., 2006. However, task behavioral analyses were repeated in a subset of individuals who meet ADI-R criteria as specified by Risi et al., 2006 (Table S1)."

Line 491 –
"The pattern of results reported here is also replicated in the additional analyses conducted with the subset of ASD individuals with meet ADI-R criteria (Text S2, Figure S2)."

The following has been added to the Supporting information (see Text S2, Line S89):

"We also conducted behavioral analyses using a subset of ASD individuals with a narrower definition of ASD as specified by Risi and colleagues (Risi et al., 2006) using the ADI-R. The criteria are as follows: meets criteria on ADI-R Social domain (A: Social reciprocity >= 10) and meets criteria on either ADI-R Communication (B: Verbal >=8) or ADI-R Behavior domain (C: Repetitive behavior >= 3). 307 of 321 ASD participants had sufficient data to calculate the Risi threshold. Of the 307 with available data, 236 met Risi criteria (77%; Table S1).

Table S1. *Participant numbers and ADI-R scores (mean, SD) for the full ASD sample and the Risi et al. (2006) ADI-R criteria subsample*

	Full ASD sample	Risi et al. ADI-R criteria subsample
N	321	236
ADI-R Social Reciprocity	15.85 (6.81)	18.47 (5.08)
ADI-R Communication	12.81 (5.64)	14.59 (4.71)
ADI-R RRB	4.25 (2.68)	4.70 (2.51)

Results are, once again, largely unchanged (see Figures S2I-L, compare to Figures S2A-D). As in the full sample, diagnostic groups did not differ on sex or age, either overall or within each age group (all ps>.1), however, all groups differed significantly on full-scale IQ, with TD groups scoring higher than ASD groups (ps ranging .026 to .0001). As in the full sample findings, repeated-measures analysis of accuracy showed significant main effects of phase ($F_{(1,481)}$=275.44, p<×10^{-16}), diagnosis ($F_{(1,481)}$=17.85, p=2.87×10^{-5}) and age group ($F_{(2,481)}$=13.54, p=1.89×10^{-6}), but no significant interactions (all ps>.1). Post-hoc analyses again showed accuracy was on average significantly higher: (i) in the acquisition phase than the reversal phase, (ii) in TD individuals compared to ASD individuals and (iii) in older age groups compared to younger age groups (Adults-Adolescents, p=.018, d=0.28; Adults-Children, p<.0001, d=0.62; Adolescents-Children, p=.018, d=0.32).
The significant main effect of diagnosis on perseverative errors was again observed ($F_{(1,480.69)}$=10.93, p=.0010, d=0.32), such that ASD individuals made on average significantly more perseverative errors than TD individuals. As before, however, there was no significant age effect nor interaction between diagnosis and age group (ps>.3).

Feedback sensitivity analysis also replicated the pattern of results in the full sample analyses: ASD individuals again showed on average significantly less win-stay and more lose-shift behavior relative to TD individuals, and for both there was a main effect of age (win-stay: diagnosis ($F_{(1,480.61)}$=9.22, p=.0025, d=0.26), age group ($F_{(2,455.25)}$=25.41, p=3.45×10^{-11}); lose-shift: diagnosis ($F_{(1,391.67)}$=9.42, p=.0023, d=0.30), age group ($F_{(2,228.42)}$=15.58, p=4.54×10^{-7}). As before, pairwise post-hoc comparisons showed win-staying increased and lose-shifting decreased with age. In these analyses, the predicted interaction between diagnosis and age group for win-stay behavior was not significant (p=.1), however, pre-planned within-age group between-diagnosis group analysis showed ASD adolescents displayed less win-staying than TD adolescents, as in the full sample analyses (p=0.0007, d=0.51). As before, this survived Bonferroni correction (correcting for task behavioral measures × age groups: p value =.05/(3×3)=.0056). For lose-shift behavior, the pattern of results was also unchanged: there was no significant interaction between diagnosis and age group (p=.4) and no between-diagnosis group age group comparisons survived Bonferroni correction (ps>.008).

To conclude, the pattern of results is largely unchanged when examining a ‘narrower’ ASD subsample (see Figure S2).”

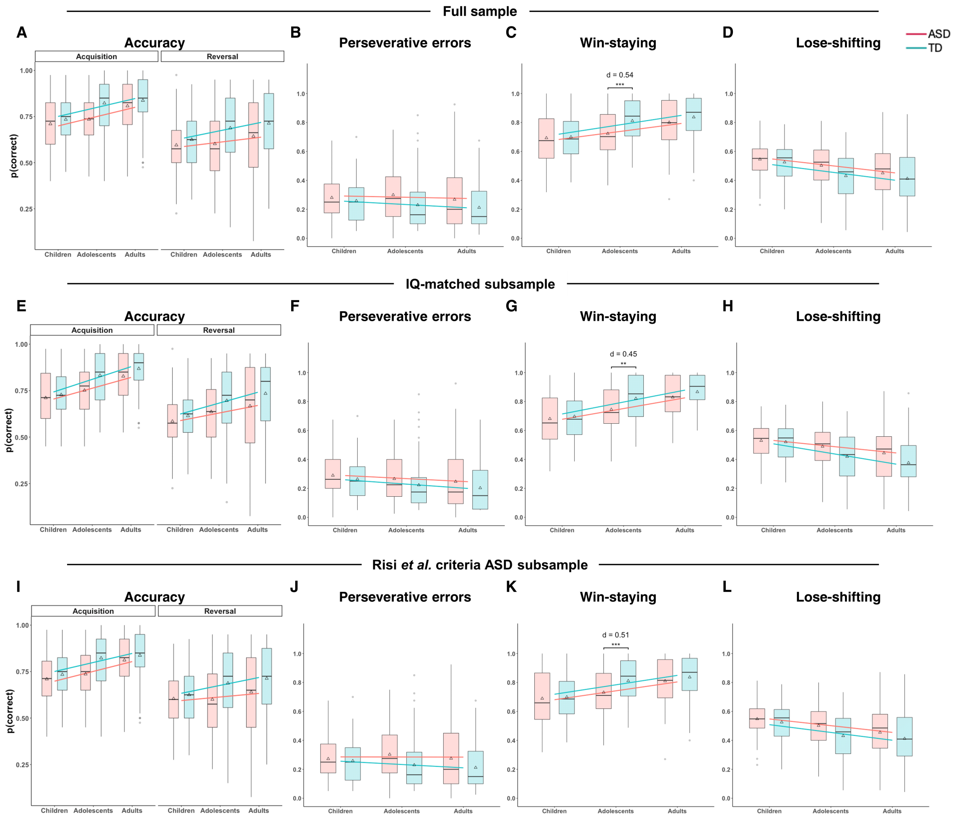

*“**Figure S2:** Boxplots showing task behavior for (A-D) the full sample, (E-H) the IQ-matched subsample and (I-L) the Risi et al. ADI-R criteria ASD subsample**.** The pattern of results remains largely unchanged across both subsample analyses.”*

2) How much were the correlations between performance and symptom severity predicted ? Is it a problem that associations are found for some clinical outcomes but not others (even though some of them arguably tap the same construct).

Response 2: We thank the reviewer for these interesting thoughts. Indeed the associations between performance and RRB symptom severity were predicted and this has been more clearly outlined in the manuscript (Line 210, see below). Furthermore, we suggest that the absence of correlations with social-communication difficulties is an important finding with respect to the specificity of cognitive flexibility to RRB. In terms of differences between symptomatology measures examining the same construct (ADI-R RRB and RBS-R), we would not consider it a problem as differences may be reflective of the clinical judgment incorporated into ADI-R scores and differences between parent-report measures and clinician-rated instruments have been reported in a number of previous studies.

“Finally, we predicted that reduced flexible behavior would be related to higher RRB symptom severity, in particular insistence on sameness/behavioral rigidities.”

3) Based on the methods section, it isn't clear to me how the ASD and TD group were matched. Do they *happen* to not differ on age and sex or were they *chosen* to match on these variables? Given that the groups differ on IQ,

I was wondering why the authors had not decided to restrict their analysis to a well-matched sample.

I do not think that the additional analyses provided in the SM address this concern appropriately. Covariance analyses assume statistical properties that are hard to meet and the analyses are hard to interpret when the groups differ substantially on the covariate, which they do. These analyses also require that the relationship between covariate and outcome be the same in both groups (ie regression slopes), which they are not (unless I missed something).

One option would be to test a larger control group (because it is comparatively harder to gather data from patients). This would allow the authors to regress performance in the RL task against IQ in the TD group and then check for each individual in the ASD group whether there is a discrepancy between observed and expected performance given observed IQ.

If that is not feasible in the current study, I would recommend random matching on age, sex and IQ (using an automated algorithm such as the matching package in R) and the subsequent analyses to be presented in the main text.

There are indeed issues with matching but given the particular topic at stake here and given the statistical assumptions behind covariate analyses, this strategy appears sounder statistically. Specifically, matching may be better recommended when the variable used to match groups really controls for a constraint on experimental task performance that isn't central to the hypothesis. My understanding is that we are in a such a situation here IQ is a predictor of learning but the hypothesis is about perseveration, not intelligence.

At the very least, given that the analyses were not pre-registered, the reader should have the option of seeing what happens when multivariate matching is done (on age sex and IQ). If the results are strikingly different, this should be discussed transparently.

Response 3: We thank the reviewer for also reflecting on the full sample IQ difference. Participants of the LEAP sample were recruited with age-, sex- and IQ-matched profiles in mind. However, recruitment challenges resulted in IQ differences between cases and controls. Following this, and as explained above in further detail (see our Response #4 to Reviewer 1, page 4), we were then interested to understand the role of IQ on task performance, thus presenting results both with and without the effects of IQ.

We have now additionally included an IQ-matched subsample analysis in the supplementary information, showing comparable results to the full sample analyses (see our Response #4 to Reviewer 1, page 4). Attempts to match groups on sex, age and IQ produced significantly limited numbers; therefore we prioritized matching groups on IQ and confirmed no differences in resulting age and IQ profiles (see

Table S3). Analyses in the IQ-matched subsample are introduced in the manuscript (Line 514) and further outlined in the supporting information (Text S2, Table S3 and Figure S2) as we think that the current scope of the main manuscript is of such a length and depth that to also present these IQ-matched sub-analyses in the main manuscript would detract from the focus of the paper, particularly because they don't change the results. However, we orient the reader to these additional analyses at the during the manuscript and have added the following to the discussion (Line 656):

"Furthermore, this pattern of results was consistent in both subsample analyses, showing robustness of findings in both an IQ-matched subsample and a subsample including only those ASD individuals who reach ADI-R criteria."

References:

Boorman, E. D., Behrens, T. E., & Rushworth, M. F. (2011). Counterfactual choice and learning in a neural network centered on human lateral frontopolar cortex. PLoS biology, 9(6).

Camerer, C., & Hua Ho, T. (1999). Experience-weighted Attraction Learning in Normal Form Games. *Econometrica, 67*(4), 827-874. doi:doi:10.1111/1468-0262.00054

Chamberlain, S. R., Müller, U., Blackwell, A. D., Clark, L., Robbins, T. W., & Sahakian, B. J. (2006). Neurochemical modulation of response inhibition and probabilistic learning in humans. *Science, 311*(5762), 861-863. doi:10.1126/science.1121218

Charman, T., Loth, E., Tillmann, J., Crawley, D., Wooldridge, C., Goyard, D., . . . Buitelaar, J. K. (2017). The EU-AIMS Longitudinal European Autism Project (LEAP): clinical characterisation. *Mol Autism, 8*, 27. doi:10.1186/s13229-017-0145-9

Cools, R., Clark, L., Owen, A. M., & Robbins, T. W. (2002). Defining the Neural Mechanisms of Probabilistic Reversal Learning Using Event-Related Functional Magnetic Resonance Imaging. *The Journal of Neuroscience, 22*(11), 4563-4567. doi:10.1523/jneurosci.22-11-04563.2002

den Ouden, H. E., Daw, N. D., Fernandez, G., Elshout, J. A., Rijpkema, M., Hoogman, M., . . . Cools, R. (2013). Dissociable effects of dopamine and serotonin on reversal learning. *Neuron, 80*(4), 1090-1100. doi:10.1016/j.neuron.2013.08.030

Gelman, A., Carlin, J. B., Stern, H. S., Dunson, D. B., Vehtari, A., & Rubin, D. B. (2013). *Bayesian Data Analysis, Third Edition*: Taylor & Francis.

Gläscher, J., Hampton, A. N., & O'Doherty, J. P. (2009). Determining a role for ventromedial prefrontal cortex in encoding action-based value signals during reward-related decision making. Cerebral cortex, 19(2), 483-495.

Hampton, A. N., Adolphs, R., Tyszka, J. M., & O'Doherty, J. P. (2007). Contributions of the amygdala to reward expectancy and choice signals in human prefrontal cortex. Neuron, 55(4), 545-555.

Ho, D., Imai, K., King, G., & Stuart, E. A. (2011). MatchIt: Nonparametric Preprocessing for Parametric Causal Inference. *2011, 42*(8), 28. doi:10.18637/jss.v042.i08

Jarrold, C., & Brock, J. (2004). To Match or Not to Match? Methodological Issues in Autism-Related Research. *J Autism Dev Disord, 34*(1), 81-86. doi:10.1023/B:JADD.0000018078.82542.ab

Lawrence, A. D., Sahakian, B. J., Rogers, R. D., Hodge, J. R., & Robbins, T. W. (1999). Discrimination, reversal, and shift learning in Huntington's disease: mechanisms of impaired response selection. *Neuropsychologia, 37*(12), 1359-1374.

Leekam, S. R., Prior, M. R., & Uljarevic, M. (2011). Restricted and repetitive behaviors in autism spectrum disorders: a review of research in the last decade. *Psychol Bull, 137*(4), 562-593. doi:10.1037/a0023341

Lesage, E., Aronson, S. E., Sutherland, M. T., Ross, T. J., Salmeron, B. J., & Stein, E. A. (2017). Neural signatures of cognitive flexibility and reward sensitivity following nicotinic receptor stimulation in dependent smokers: a randomized trial. *JAMA psychiatry*, *74*(6), 632-640.

Lord, C., Risi, S., DiLavore, P. S., Shulman, C., Thurm, A., & Pickles, A. (2006). Autism from 2 to 9 years of age. *Arch Gen Psychiatry, 63*(6), 694-701. doi:10.1001/archpsyc.63.6.694

Lord, C., Risi, S., Lambrecht, L., Cook, E. H., Leventhal, B. L., DiLavore, P. C., . . . Rutter, M. (2000). The Autism Diagnostic Observation Schedule—Generic: A Standard Measure of Social and Communication Deficits Associated with the Spectrum of Autism. *J Autism Dev Disord, 30*(3), 205-223.

Lord, C., Rutter, M., DiLavore, P. C., Risi, S., Gotham, K., & Bishop, S. (2012). *Autism Diagnostic Observation Schedule, Second Edition (ADOS-2) Manual (Part I): Modules 1–4*. Torrance, CA: Western Psychological Services.

Murphy, F. C., Michael, A., Robbins, T. W., & Sahakian, B. J. (2003). Neuropsychological impairment in patients with major depressive disorder: the effects of feedback on task performance. *Psychol Med, 33*(3), 455-467.

Risi, S., Lord, C., Gotham, K., Corsello, C., Chrysler, C., Szatmari, P., . . . Pickles, A. (2006). Combining information from multiple sources in the diagnosis of autism spectrum disorders. *J Am Acad Child Adolesc Psychiatry, 45*(9), 1094-1103. doi:10.1097/01.chi.0000227880.42780.0e

Rutter, M., Le Couteur, A., & Lord, C. (2003). *Autism Diagnostic Interview-Revised*. Los Angeles, CA: Western Psychological Services.
