# Supplementary material for: Modeling flexible behavior in childhood to adulthood shows age-dependent learning mechanisms and less optimal learning in autism in each age group
Source: PLoS Biol. 2020 Oct 27;18(10):e3000908. doi: 10.1371/journal.pbio.3000908 (PMC7591042; doi:10.1371/journal.pbio.3000908)
Supplement: Attachment — Submitted filename: PRL_Response to reviewers comments_2b_DC_LZ.pdf [file pbio.3000908.s024.pdf]

Friday, September 18, 2020

Response to Reviewers' comments

Manuscript ID: PBIOLOGY-D-19-03505R2

Current manuscript title: "Modeling flexible behavior in children, adolescents and adults with autism spectrum disorder and typical development"

Title change: We would like you to consider changing your title, which we think is too descriptive. We recommend one that conveys the central biological message and suggest the following. However, we are open to discuss alternatives: "Modeling flexible behavior deficits in Autism Spectrum Disorder shows age-dependency and less optimal learning within each age group".

We recognise the usefulness of conveying the paper's main message in the title. We are largely happy with the suggestion; however, we would prefer not to use the term 'deficits'. We therefore suggest:

"Modeling flexible behavior from childhood to adulthood shows age-dependent learning mechanisms and less optimal learning in autism in each age group"

We have also edited the running title to "autism" rather than "autism spectrum disorder" to reflect some preferences within the autistic community not to use the term 'disorder'. We have made these changes accordingly in the manuscript in the hope that you find these suggestions agreeable. However, we are also open to discussing alternatives.

ETHICS STATEMENT:

-- Please include the full name of the IACUC/ethics committee that reviewed and approved the animal care and use protocol/permit/project license. Please also include an approval number.

-- Please include the specific national or international regulations/guidelines to which your animal care and use protocol adhered. Please note that institutional or accreditation organization guidelines (such as AAALAC) do not meet this requirement.

-- Please include information about the form of consent (written/oral) given for research involving human participants. All research involving human participants must have been approved by the authors' Institutional Review Board (IRB) or an equivalent committee, and all clinical investigation must have been conducted according to the principles expressed in the Declaration of Helsinki.

We can confirm that this research was approved by each site/country's IRBs or equivalent. As the EU-AIMS Longitudinal European Autism Project (LEAP) was conducted in several sites/countries, each country applied for ethics to their respective review boards. Written informed consent was obtained from all participants or their legal guardian. This was noted in the Methods section. The full details, including confirmation that the research was conducted according to the Declaration of Helsinki principles, are now listed in the Ethics Statement (Page 8, Line 164):

"The study was approved by the independent local ethics committees of the participating centers (London Queen Square Health Research, Authority Research Ethics Committee: 13/LO/56; Radboud University Medical Centre Institute Ensuring Quality and Safety Committee on Research Involving Human Subjects Arnhem-Nijmegen: 2013/455; UMM University Medical Mannheim, Medical Ethics Commission II: 2014-540 N-MA; University Campus Bio-Medical Ethics Committee of Rome: 18/14 PAR ComET CBM) and conducted according to the principles expressed in the Declaration of Helsinki. Written informed consent was obtained from all participants and/or their parent/guardian (where appropriate) prior to the study."

DATA POLICY:

We note that you have stated in the online submission system that readers can access the raw data by contacting the EU-AIMS LEAP group. However, we request that you provide the underlying numerical values that underlie the summary data displayed in the following figure panels: Figures 1C, 2ABCDE, 3CD, 4A-J, S1, S2A-L, S3AB, S4, and S7.

The numerical data provided should include all replicates AND the way in which the plotted mean and errors were derived (it should not present only the mean/average values).

We have collated the numerical data underlying the figures and figure panels listed above. We have included these in an excel sheet entitled 'S1 Data', with separate sheets referring to specific figures/figure panels.

Reviewer #2:

1/ In response to my point R2.1 (counterfactual learning rate issue) the authors mention that (quote):

"we would like to point out that our method of model comparison does indeed account for this increased functional complexity in the counterfactual update model, unlike simpler methods based on e.g. AIC/BIC comparison. This is one of the motivations from our perspective to conduct all computational modeling analyses in the Bayesian framework, where inferences are drawn from joint posterior

distributions, rather than point estimates. Under the Bayesian framework, the joint parameter spaces of the CU and the simple RL model differ; thus, the effective number of parameters of these two models is different. It is the latter (i.e., effective number of parameters) that we use in the penalizing term in computing the model evidence"

However, it is still unclear to me how it is possible that functional complexity (i.e., adding one additional equation - or complexifying it) is taken into account for penalization in their Bayesian approach. As this is a crucial issue (and their claim is counterintuitive), I believe that the authors should unpack and explain this point better in the revised manuscript.

As indicated in our last response letter, the Bayesian MCMC approach we used here considers the joint parameter space, which is characterised by the model equations. The additional equation in the CU model (relative to the RW model) changes the complexity of the parameter space. In fact, the effective number of parameters of CU is consistently larger than that of the RW model (see Table below), which is otherwise not detectable using methods like LME. We have now added the following text to S1 Text ('Supplementary Methods'), Page ii, Line 49:

"Although it appears that the number of parameters of the RW and CU models are identical, the additional equation in the CU model actually changes the joint parameter space, resulting in more effective number of parameters than the RW model (Table S2).

Table S2: Effective number of parameters of RW and CU models

		RW	*CU*
TD	*Children*	*78*	*104*
	Adolescents	*125*	*161*
	Adults	*137*	*176*
ASD	*Children*	*101*	*133*
	Adolescents	*154*	*204*
	Adults	*200*	*246*

2/ In response to my point R2.2 the authors decided to maintenant the name EWA for their model, based on the fact that they used the same name in a previous paper (2013), while they acknowledge that their model lacks the distinctive features of the EWA model. I still think that the label is not appropriate and, in a sense, "historically unfair". I strongly encourage the authors to take a look at Table 6.2 and table 6.3 of the book "Behavioral Game Theory" (by Colin Camerer), they will see their model will be classified as "Reinforcement Learning" (Roth and Erev) by Camerer himself.

Let's say a first group invent a model with the process X inside and call it 'John'. A second group invent a model with the processes X and Y inside and call it 'Paul'. A third group uses a model with the process X

inside and call it 'Paul with no Y'. The first group would have all the rights to be annoyed, right? Of course this is not such a big deal, but I would love to know what are the authors' thoughts on this.

In the original Neuron paper we labelled this model EWA to pay homage to the model where we took the equations for nonstationary updates with time. Clearly it is not the same model as the original EWA model by Camerer and Ho, which it cannot be as it is not the same class of tasks. The original EWA has two important defining dimensions: it is most famous for blending what they called belief learning vs. reinforcement learning (RL) – we would now term this model-based (MB) and model-free learning (MF; the 'delta' parameter). The other dimension is the averaging vs. accumulating / learning rate nonstationarity property (the 'rho' parameter). To our knowledge, the latter dynamical learning rate involving rho is not something that was taken from earlier reinforcement learning models (as indicated in Camerer Table 6.2), but rather this is the form that EWA introduced to interpolate between different forms of updating (accumulating versus averaging when rho shifts from 0 to 1). We had assumed – both here and in our original paper – that the 'experience weight' refers to exactly this parameter, rho (and the experience weight that accumulates as a function of rho), hence our naming of this model.

However, perhaps it is true that people mainly think the crucial difference and innovation of EWA is delta (the MB vs. MF dimension) and it is what readers may think of when they see the reference to EWA. Indeed we do not have this feature in our model, because MB vs. MF are simply identical in this task and we can't distinguish them. Thus, the model that we are using is in fact a reduced EWA model that does not distinguish the belief learning vs. RL because the task was not designed to.

That said we are happy to use a different name if the reviewer thinks it may cause readers to expect something that we are not modelling. However, we are challenged to think of a better name, because the defining feature is to use the accumulating experience weight eta (named thus in the original paper). We do not want to simply call it a 'dynamic learning rate model', given the possibility of confusion with Pearce-Hall and Hierarchical Gaussian Filter models (amongst others). In all, we would prefer to stick with EWA as this is where our experience weighted dynamic learning rate feature comes from, but could be happy with a compromise of "EWA dynamic learning rate".

We have now updated the model labelling, however, we are open to further suggestions from the reviewer and editor.

We have also clarified in the methods section the history of this model, adding in the following footnote when first introducing the model (Page 13, Line 284):

"In our previous paper [47] we labeled this model EWA to pay homage to the model where we took the equations for nonstationary updates with time. However, we do not use the exact same model as the original EWA [55]. We do not include the feature of blending belief learning' versus reinforcement

learning (now more commonly termed model-based, MB, and model-free, MF, learning) indexed by the delta δ parameter. Rather, we only use the rho ρ parameter that allows for a nonstationary learning rate through updating of an experience weight. This dynamical learning rate allows for interpolation between different forms of updating (accumulating versus averaging who rho shifts from 0 to 1). We used the EWA label for this reduced EWA model in our original paper, as (we assumed) the 'experience weight' label referred to this model feature. However, we have become aware that for many readers, the crucial innovation of EWA is the delta δ parameter that allows for the mix of MB and MF updating. We have therefore relabeled this model as EWA-dynamic learning rate to make this distinction clear. Finally, note that we omitted the delta feature from our model because this task cannot distinguish MB vs. MF learning."

3/ in the SI 'Additional methods' I believe the authors wanted to cite Lefebvre 2017, rather than 2018.

We have indeed amended this citation to the 2017 paper in Text S1, page iv, line 127:

"These findings contribute to continued debates within the literature regarding higher learning rates for positive value compared to negative value stimuli (see also [12-14]).

…

13. Lefebvre G, Lebreton M, Meyniel F, Bourgeois-Gironde S, Palminteri S. Behavioural and neural characterization of optimistic reinforcement learning. Nature Human Behaviour. 2017;1(4). doi: 10.1038/s41562-017-0067."
